# Genetic and morpho-physiological analyses of the tolerance and recovery mechanisms in seedling stage spring wheat under drought stress

**DOI:** 10.3389/fgene.2022.1010272

**Published:** 2022-10-11

**Authors:** Asmaa A. M. Ahmed, Mona F. A. Dawood, Ameer Elfarash, Elsayed A. Mohamed, Mohamed Y. Hussein, Andreas Börner, Ahmed Sallam

**Affiliations:** ^1^ Department of Genetics, Faculty of Agriculture, Assiut University, Assiut, Egypt; ^2^ Department of Botany and Microbiology, Faculty of Science, Assiut University, Assiut, Egypt; ^3^ Resources Genetics and Reproduction, Department Genebank, Leibniz Institute of Plant Genetics and Crop Plant Research (IPK), Gatersleben, Germany

**Keywords:** drought tolerance, genetic variation, morphological traits, seedling stage, spring wheat, physiological traits, DREB genes

## Abstract

Drought is one of the complex abiotic stresses that affect the growth and production of wheat in arid and semiarid countries. In this study, a set of 172 diverse spring wheat genotypes from 20 different countries were assessed under drought stress at the seedling stage. Besides seedling length, two types of traits were recorded, namely: tolerance traits (days to wilting, leaf wilting, and the sum of leaf wilting), and recovery traits (days to regrowth, regrowth biomass, and drought survival rate). In addition, tolerance index, recovery index, and drought tolerance index (DTI) were estimated to select the most drought tolerant genotypes. Moreover, leaf protein content (P), amino acid (AM), proline content (PRO), glucose (G), fructose (F), and total soluble carbohydrates (TSC) were measured under control and drought conditions to study the changes in each physiological trait due to drought stress. All genotypes showed a high significant genetic variation in all the physio-morphological traits scored under drought stress. High phenotypic and genotypic correlations were found among all seedling morphological traits. Among the studied indices, the drought tolerance index (DTI) had the highest phenotypic and genotypic correlations with all tolerance and recovery traits. The broad-sense heritability (H^2^) estimates were high for morphological traits (83.85–92.27), while the physiological traits ranged from 96.41 to 98.68 under the control conditions and from 97.13 to 99.99 under drought stress. The averages of the physiological traits (proteins, amino acids, proline, glucose, fructose, and total soluble carbohydrates) denoted under drought stress were higher than those recorded under well-watered conditions except for proteins. In this regard, amino acids, glucose, and total soluble carbohydrates had a significant correlation with all morphological traits. The selection for drought tolerance revealed 10 tolerant genotypes from different countries (8 genotypes from Egypt, one from Morocco, and one from the United States). These selected genotypes were screened for the presence of nine specific *TaDREB1* alleles. Six primers were polymorphic among the selected genotypes. Genetic diversity among the selected genotypes was investigated using 21,450 SNP markers. The results of the study shed light on the different mechanisms for drought tolerance that wheat plants use to tolerate and survive under drought stress. The genetic analysis performed in this study suggested the most suitable genotypes for selective breeding at the seedling stage under water deficit.

## Introduction

Wheat (*Triticum aestivum* L.) is one of the most important cereal crops in the world. The main losses in wheat production are due more to abiotic stresses such as drought (Ballesta et al., 2020; Ahmad et al., 2022), salinity (Yousfi et al., 2016), and high temperatures (Posch et al., 2019) than biotic stresses. Drought stress affects plant development, growth, and crop production, especially in arid and semi-arid countries (Moursi et al., 2020). In 2013, approximately 65 million hectares of wheat production was affected by drought stress (Boliko, 2019). Drought stress can occur at any growth stage (germination, seedling, vegetative, flowering, and reproductive) depending on the local environment, and it affects almost every aspect of plant growth through alterations in metabolism and gene expression (Sallam et al., 2019). Early growth stages, such as the seedling stage, are critical and very sensitive stages to drought stress because they affect all the following stages, including grain yield (Samarah, 2005). Consequently, studying drought tolerance at these stages is very important to increase the selection efficiency for drought-tolerant varieties in the breeding programs (Hameed et al., 2010). With the consequences of climate change, the severity of drought stress is expected to increase at any growth stage, especially the seedling stage, which is fundamental to plant architecture and development.

Breeding for drought tolerance is a crucial solution to producing cultivars with high drought tolerance. Therefore, wheat breeders and geneticists aim to address the variation in drought tolerance by scoring new traits directly associated with drought tolerance. (Ehdaie et al., 1991; Ehdaie and Waines, 1993; Mwadzingeni et al., 2017). Various morphological traits have been used to measure the effect of drought on plant leaves at the seedling stage. Examples include, but are not limited to, leaf wilting, days to wilting, the sum of leaf wilting, regrowth biomass, and days to regrowth (Sallam et al., 2018b; Ahmed et al., 2021). These traits are very useful because they discriminate between tolerant and susceptible genotypes. Moreover, they measure the ability of plants to tolerate prolonged water shortages. In addition, they measure the plant’s ability to recover after drought exposure. Therefore they are very effective for selection in a breeding program to improve drought tolerance (Ahmed et al., 2021). A previous study by Sallam et al. (2018b) reported two types of traits recovery (regrowth) and tolerance in winter wheat under drought at the seedling stage. They found no correlation between the recovery and tolerance traits but a highly significant correlation among traits within each type. However, no physiological analyses were reported. Here, we applied the same protocol suggested by Sallam et al. (2018b) in a highly diverse spring wheat core collection (WCC) collected from 20 countries to investigate this relationship in the spring type.

In addition to morphological alterations by drought, there are many physiological changes that wheat plants make to withstand the effect of drought stress. The physiological changes due to drought stress differ by the growth stage and also by the genotype (Farshadfar et al., 2008; Sallam et al., 2019). The most important biochemical attributes that are widely accepted as fundamental traits related to drought stress are water content (Abid et al., 2018), proline (Ahmad et al., 2015; Mwadzingeni et al., 2016), chlorophyll content (Nikolaeva et al., 2010; Allahverdiyev et al., 2015), amino acids content (Abid et al., 2016), and photosynthesis efficiency (Ahmad et al., 2018). It was reported that tolerant genotypes tend to accumulate soluble sugars, accumulate amino acids, increase chlorophyll content in leaves, reduce the rate of water loss, reduce photosynthetic activity, and increase its proline content (Zali and Ehsanzadeh, 2018). Thus, evaluating the plant physio-morphological traits is very important for selection to improve drought tolerance in a breeding program due to their relation to the adaption for future climate scenarios (Bowne et al., 2012). Physiological analyses provide helpful information on understanding the mechanisms in plants to alleviate the effect of drought stress (Sallam et al., 2018a; Dawood et al., 2020; Mondal et al., 2021; Moursi et al., 2021). Such information can be used along with morphological traits for selecting the most drought-tolerant cultivars with high adaptability to drought stress at the seedling stage. Moreover, to validate the selection results, screening the selected tolerant genotypes using DNA molecular markers for specific drought genes such as dehydration-responsive element-binding protein (*DREB*) gene is highly recommendable to select the target candidate’s parents for future crossing in breeding programs. Also, crossing highly genetically diverse drought-tolerant genotypes will be fruitful in producing cultivars with a high drought tolerance level.

Thus, the objectives of the current study were to 1) assess the genetic variation in tolerance and recovery traits of a highly diverse spring wheat core collection, 2) understand the essential physiological changes under drought stress, and 3) select the most promising spring wheat genotypes with high drought tolerance at the seedling stage for the future breeding program.

## Materials and methods

All experiments and activities conducted in this study were illustrated in Supplementary Figure S1.

### Plant material

The plant material consisted of 172 highly diverse spring wheat genotypes (Supplementary Table S1) and two checks; Wesley (a drought susceptible cultivar) and Harry (a drought tolerant cultivar) (Hussain et al., 2018; Sallam et al., 2019). Out of the 172 genotypes, 20 were from Egypt, while the remaining 152 genotypes were from 19 different countries. These genotypes represent 20 different countries covering wide geographic regions around the world, including Egypt, Afghanistan, Algeria, Australia, Canada, Ethiopia, Germany, Greece, Iran, Kazakhstan, Kenya, Morocco, Oman, Saudi Arabia, Sudan, Syria, Tunisia, United Kingdom, and the United States. Moreover, the genotypes represented the following continents: Africa, Asia, Europe, North America, and Australia and were collected from the U.S National Plant Germplasm, United States Department of Agriculture, United States. The number of genotypes used from each country is presented in Supplementary Figure S2. These genotypes showed good performance with high adaptation to the Egyptian environmental condition (Ahmed Sallam, personal communication).

### Drought assessment

#### Assessment of morphological traits at the seedling stage

##### Experimental layout

Drought experiments were conducted in the Plant Genetics Lab, Faculty of Agriculture, Assiut University. The drought stress was applied to all genotypes based on the protocol described by Sallam et al. (2018b) with few modifications (drought stress period). The experimental layout was a randomized complete block design (RCBD) with seven replications. A set of 84 Cell Plant Tray (65 × 37 cm) was used. Each cell was filled with 50 g of fertilized sand soil. In each replication, four seeds from each genotype were sown in 2 cells with two seeds/cell. A final of 28 grains/genotypes were scored. The sand filtering process was used to calculate the volume of water used for each irrigation (Supplementary Figure S3A,B).

All genotypes grown in tray cells were firstly irrigated with 16 ml distilled water (100% soil water capacity). Then, all genotypes were irrigated with 8 ml in the second irrigation (50% soil water capacity) to prepare the genotypes for drought stress. The temperature and humidity data during the experiment were recorded daily (Supplementary Figure S4). The temperature ranged from 20 to 23 °C, and air humidity ranged from 37 to 56.6%. When the first leaf emerged (seedling emergence) after 7 days from sowing, the drought treatment was applied by water withholding. The drought treatment was stopped when 70% of plants were fully wilted (after 13 days), thus, all plants of each genotype remained without water for 13 days.

##### Traits scoring

The seedling length (SL), tolerance (TT) traits, and recovery (RT) traits were recorded on each plant as described by Sallam et al. (2018b). At the end of drought treatment (after 13 days from water withholding), the shoots (leaves and stem) of all plants/genotype were cut at the soil surface and then irrigated to test their ability to regrow after prolonged drought stress. The time from the cutting the plants to the end of experiment was 17 days as we did not observe any regrowth after that. The experiment lasted for 37 days.

1) Seedling length (SL) was measured (for each genotype) in centimeters (cm) from the beginning of the soil surface to the end of the plant. This trait was scored before water withholding (before drought stress).
2) The tolerance traits (TT) included:
  A) Days to wilting (DTW) was scored as the number of days from starting water withholding until 50% of seedlings/genotype started to wilt. High values indicated tolerance to drought stress.
  B) Leaf wilting (LW) was visually scored on each seedling/genotype during drought treatment when the plants started to wilt and scored every 2 days using a scale ranging from 1 (no wilting) to 9 (fully wilted). The wilting degree as a visual score was recorded as previously described by Ahmed et al. (2021). The total visual scores of LW from the start of withholding water until the end of drought treatment (during the entire drought duration (13 days)) were done five times. Low values indicated high tolerance to drought stress.
  C) Sum of leaf wilting (S_LW). The five scores of LW were summed up to form one trait to evaluate the wilting symptoms for each genotype during the drought period. This trait ranged from 5 (no wilting) to 45 (fully wilted). Low values indicated high tolerance to drought stress.
3) The recovery traits (RT) included:
  A) Days to Regrowth (DTR) was determined for each seedling after their cutting (shoots), and this trait was counted as the number of days from the beginning of cutting plants (shoots) until the regrowth of plants where each uprooted seedling started to produce the first new leaf. This trait estimated the ability of cut plants to produce new shoots after exposure to 13 days of drought stress when re-watered. Low values of DTR indicated high tolerance to drought stress. All scores of DTR traits were converted or transformed to the disposition to regrowth [from 0 to 90] as described by both Roth and Link (2010); Sallam et al. (2018b) according to the following equation:

$$DTR=\arctan_{xi}/\mu_{x}$$

where x_i_ number of days for each cutting plant (leaves) from the beginning of cutting until the production of the first new shoot, µ_x_ = average number of days for those plants that produced new shoot after re-watering. Plants that cannot form or produce a new shoot after the drought was considered to be lifeless plants and had a score of 90.

B) Regrowth biomass (RB) was scored for each regrowth plant on the last day of the experiment by re-cutting the leaves and shoots of plants that regrowth after drought stress when re-watering and weighed (g). High values indicated high tolerance to drought stress.

C) Drought survival rate (DSR) was estimated in each replication for each genotype by calculating the number of surviving plants from cut plants (number of plant/genotypes = 4) by dividing the number of surviving plants from cut plants to the number of cut plants where high values of DSR indicated tolerance to drought stress.

$$DSR=\frac{No.\;of\;surviving\;plants}{No.\;of\;cut\;plants}\times 100$$

Selection index for drought tolerance.

Three selection indices were calculated, as shown in Falconer and Mackay (1996), to better select or determine the most drought-tolerant genotypes (Falconer, 1996).

The tolerance index (TI), which represented the tolerance traits and was used to better describe S-LW (X_1_) using two auxiliary traits: DTW (X_2_) and SL (X_3_) as:

$$TI=b_{1}X_{1}+b_{2}X_{2}+b_{3}X_{3}$$

Where, b_1_ = 0.7429, b_2_ = -0.3808, b_3_ = 0.0186.

Recovery Index (RI), which represented recovery traits and was used to better describe DTR (X_1_) using two auxiliary traits: RB (X_2_) and DSR (X_3_) as:

$$RI=b_{1}X_{1}+b_{2}X_{2}+b_{3}X_{3}$$

Where, b_1_ = 0.6424, b_2_ = -0.1389, b_3_ = -0.0267.Where b_1_, b_2_, and b_3,_ b_4,_ are the index coefficients. The vector of Smith-Hazel index coefficient b was calculated as shown in Baker (1986).b = P ^−1^ G, where P ^−1^ is the inverse of the phenotypic variance-covariance matrix for the traits; G is a matrix including the estimates of genotypic and covariance.

Drought Tolerant Index (DTI) was calculated by combining both TI and RI as follow:

DTI=[(TI/SDTI)+(RI/SDRI)]1/2

Where SD_TI_ and SD_RI_ are the phenotypic standard deviation of the TI and RI, respectively. The low DTI values indicated high tolerance to drought stress.

### Assessment of physiological traits at the seedling stage

At the end of the drought experiment, the shoot of each genotype across the seven replications was dried and pooled for physiological analysis. Along with this, the same genotypes were sown in three replications under control conditions (normal irrigations) and after 13 days, the leaves were cut and dried. The shoot dry matter for each genotype under both treatments was used for assessing the different physiological parameters. Six physiological traits were estimated, protein content (PC), total soluble carbohydrates (TSC), glucose (G), fructose (F) and amino acid (AM) contents, and proline content (PRO).

1) Protein content (P)

The protein content of the aqueous extract was determined using an alkaline reagent solution according to the method of Lowery et al. (1951) where the Folin solution was used as an indicator for protein detection.

2) Total soluble carbohydrates (TSC)

Glucose (G) and fructose (F) mg/g DW were estimated in the aforementioned extract using the anthrone-sulfuric acid method for both Halhoul and Kleinberg (1972), while the total soluble carbohydrate (TSC) in the same extract (mg/g DW) was estimated by False (1951).

3) Amino acids (AM)

The ninhydrin method described by Moore and Stein (1948) was followed to estimate the total amino acid in leaves, and a diluent Solvent was used as standard.

4) Proline content (PRO)

To estimate the proline content in the leaves, an extract was made by grinding dried leaves (0.05 g) in 3 ml of 5% sulfosalicylic acid. The extract was filtered, and the supernatant was used to determine the proline following the method by Bates et al. (1973).

### Statistical analysis of the phenotypic data

The analysis of variance, covariance, boreas-sense heritability, Spearman rank correlation, and genotypic correlation were perfomed using PLABSTAT software (Utz, 1997). Two statistical models were used. First model was used to analyze the morphological traits scored under drought stress using the following model.

$$Yij=\mu +g_{i}+r_{j}+gr_{ij}\left(error\right)$$

Where *Yij* is the observation of genotype *i* in replication *j*, *µ* is the general average, *gi* and *rj* are the main effects of genotypes and replication, respectively, and the error is the interaction between genotype *i* and replication *j*. For seedling data, genotypes and replications were considered random effects. Broad-sense heritability (H^2^) estimates for each trait were calculated by PLABSATA using the following equation:

$$H^{2}=\frac{\sigma_{G}^{2}}{\sigma_{G}^{2}+\left(\sigma_{GR}^{2}\right)}$$

where $\sigma_{G}^{2}$ refers to genotypic variance, while $\sigma_{G}^{2}+\left(\sigma_{GR}^{2}\right)$ refers to the phenotypic variance.

Second, another statistical model was used to analyze the physiological traits that were measured under control and drought stress using the following model

$$Y_{ijk}=\mu +g_{i}+r_{j}+t_{k}+tg_{ki}+tgr_{ijk}$$

where *Y*_*ijk*_ is the observation of genotype i in replication j in treatment k (control vs. drought), k, μ is the general mean; *g*_*i*_, *r*_*j*_, and *t*_*k*_ are the main effects of genotypes, replications, and treatments, respectively. *tg*_*ik*_ is genotype × treatment interaction. *tgr*_*ijk*_ is genotype × replications × treatment interaction (error). Treatments were considered fixed effects, while replications and genotypes were considered random effects.

The Spearman rank correlation coefficient was imputed by PLABSTAT to estimate the phenotypic correlation between traits. The genetic correlation coefficient was estimated for all traits using covariance analysis and GENOT-a command with PLABSTAT software, to allow the construction of optimum selection indices. Microsoft Office Excel 2010 and R software (R Core Team, 2014) were used to make some graphical of the results of the analysis, such as a histogram to show the normal distribution of genotypes on traits.

The change (increase or reduction) in each trait due to drought stress was calculated for all physiological traits that were scored in this study based on the average of each trait using the following equations for Sallam et al. (2018a). If the mean of the trait for all genotypes under control conditions are higher than the mean under drought stress, then the reduction due to drought stress in the trait (RDD) was calculated according to the following equation:

$$RDD-trait=\left(\frac{XC-XD}{XC}\right)x\;100$$

If the mean of the trait for all genotypes under drought stress is higher than the mean under control conditions, then the increase in the trait due to drought stress (IDD) was calculated according to the following equation:

$$IDD-trait=\left(\frac{XD-XC}{XD}\right)x\;100$$

where XD and XC are the means of a trait for each genotype under drought stress and control conditions, respectively.

### Genetic analysis of the most drought-tolerance genotypes

#### Screening the most drought-tolerance genotypes with specific *DREB* genes

The most drought-tolerant genotypes (N = 10) were selected, and DNA was extracted from two to three leaves of six old seedlings. The DNA extraction was performed in the Biotechnology laboratory in the Genetics Department, Faculty of Agriculture, Assiut University. DNA was extracted from each genotype from two to three leaves using the Thermo Scientific GeneJET Plant Genomic DNA Purification Mini Kit protocol. Nine primer combinations of dehydration-res element binding proteins (nine fixed forward primers in combination with nine reverse primers) developed by Liu et al. (2018) were tested with the ten drought–tolerance genotypes. Primer codes and sequences of the forward and reverse primers are shown in Table 1. A gradient PCR was performed in order to determine the optimal annealing temperature for each primer used. The gradient test was performed using a gradient annealing temperature of 70 < 60 >50. The method of Thermo scientific PCR Master Mix protocol (Scientific, 2012) was followed for PCR reactions; each amplification reaction was carried out in a total volume of 20μL, containing 1x PCR reaction mix buffer (10 μL), 0.2 of each forward and reverse primer and 1 μL of template DNA, 8.6 μL H_2_O. Polymerase chain reactions (PCRs) were carried out using the following program: initial denaturation at 94°C for 5 min, 35 cycles, while denaturation at 94°C for 30 s, annealing at 52–69.6°C for 30 s, 72°C for 60 extensions, and a final extension at 72°C for 5 min. The PCR products of each reaction (20 µl) and a 1,000 bp ladder marker (1 µl) were electrophoresed onto submerged agarose gel of 1% concentration containing 0.05 μL Supper Saffa in 50 ml TBE buffer (50X). Electrophoresis was carried out under a constant voltage of around 80 V for approximately 2–2.5 h. The banding patterns were visualized under Transilluminator and photographed using a gel documentation system (Ultra-Violet Product, Upland, CA, USA).

**TABLE 1:** Primer codes and sequences of the forward (F) and reverse (R) primers used in the *DREB* analysis.

Codes	Primer	Primer sequence	Fragment size (bp)	Anneal temp. (°C)
***DREB1-A***	F	ATG​AAC​AGG​AAG​AAG​AAA​GTG​CGC	593	62
	R	TTC​TCA​AAT​CAT​TGC​TCA​CT TCTTTC		
***DREB1-A1***	F	CGG​AAC​CAC​TCC​CTC​CAT​CTC	1,107	58.8
	R	CGG​TTG​CCC​CAT​TAG​ACG​TCA		
***DREB1-A2***	F	CTG​GCA​CCT​CCA​TTG​CTG​AC	599	67.4
	R	AGT​ACA​TGA​ACT​CAA​CGC​ACA​GGA​CAA​C		
***DREB1-B***	F	CCC​AAC​CCA​AGT​GAT​AAT​AAT​CT	816	58.8
	R	TTG​TGC​TCC​TCA​TGG​GTA​CTT		
***DREB1-B***	F	ATG​ACC​AGG​AAG​AAG​AAA​GTG​CGC	585	60
	R	TCA​TTG​CTC​ACT​TCT​TTT​TTC​ACC​TTA​T		
***DREB1-D***	F	ATG​AAC​AGG​AAG​AAG​AAA​GTG​CGC	455	52
	R	TCC​TTC​CCA​TCA​GAA​GGA​TGT​GAC		
***DREB1-D1***	F	TCG​TCC​CTC​TTC​TCG​CTC​CAT	1,190	69.6
	R	GCG​GTT​GCC​CCA​TTA​GAC​ATC​G		
***DREB1-D2***	F	CTG​GCA​CCT​CCA​TTG​CCG​AT	596	69.6
	R	AGT​ACA​TGA​ACT​CAA​CGC​ACA​GGA​CAA​C		
***DREB U***	F	TCG​TCC​CTC​TTC​TCG​CTC​CAT​GG	493	66
***DREB D***	R	GGGCATGGCG CCGCATGG		

Where (F), (R) indicates that the sequence of the forward and reverse primers, respectively.

### Genotyping-by-sequencing (GBS) and genetic diversity among the selected genotypes

The DNA of the most drought-tolerant genotypes selected in this study was sent to Trait Genetics for GBS using a 25 K wheat Infinium array at Trait Genetics, Gatersleben, Germany. Extensive details on the development of the 25 K wheat Infinium array were reported in Soleimani et al. (2020). The result of array genotyping revealed 21,450 SNP markers that were used for calculating genetic distance among the selected genotypes using R-package ‘ade4’ (Lobry et al., 2012). The genetic distance was calculated using a simple matching coefficient.

## Results

### Genetic variation at the seedling stage under drought stress

#### Genetic variation analysis of the morphological traits

Analysis of variance (ANOVA) results for the morphological (tolerance and recovery) traits scored in this study at the seedling stage are presented in Table 2 and Supplementary Table S2. The results showed a highly significant variation (*p* < 0.01) among the genotypes for all traits measured under drought stress. For the tolerance traits, the highest and lowest *p-values* among the genotypes were for LW5 and LW1, respectively. For the recovery traits, RB (11.52**, *p* > 0.01) had the highest *p-value*, while DSR (6.24**, *p* > 0.01) had the lowest. All traits had a wide range of heritability (H^2^), and the estimates for the tolerance traits ranged from 69.02 (LW1) to 87.76 (LW5), while it ranged from 83.98 (DSR) to 91.32 (RB) in recovery traits. The heritability estimates for the recovery traits were higher than those for the tolerance traits. The drought tolerance index (including TI and RI) had the highest H^2^ among the selection indices at 90.71, while seedling length (SL), which was scored before the drought, had an H^2^ value of 92.27.

**TABLE 2:** Descriptive statistics and F-values among genotypes for all morphological traits scored at the seedling stage.

Tait	Mini	Max	Mean	LSD	*F-value*	SD	H^2^
**Seedling length (SL)**	8.18	22.55	15.75	2.01	12.94**	2.602	92.27
**Tolerance traits**						
**Leaf wilting1 (LW1)**	1	2.1	1.32	0.33	3.23**	0.213	69.02
**Leaf wilting2 (LW2)**	1.22	3.52	2.39	0.7	3.98**	0.501	74.85
**Leaf wilting3 (LW3)**	2.19	5.12	3.87	0.93	3.47**	0.621	71.2
**Leaf wilting4 (LW4)**	3.69	6.65	5.45	0.9	3.49**	0.603	71.35
**Leaf wilting5 (LW5)**	5.17	8.73	7.5	0.71	8.17**	0.731	87.76
**Sum of leaf wilting (S_LW)**	13.82	25.08	20.53	2.52	6.64**	2.334	84.93
**Days to wilting (DTW)**	4.38	8.06	5.68	0.83	6.19**	0.744	83.85
**Recovery traits**						
**Days to regrowth (DTR)**	35.67	90	78.5	11.83	8.03**	12.075	87.55
**Regrowth biomass (RB)**	0	126.14	11.98	15.38	11.52**	18.808	91.32
**Drought survival rate (DSR)**	0	93.75	19.6	24.3	6.24**	21.862	83.98
**Drought indices**						
**Tolerance index (TI)**	7.42	17.18	13.38	2.04	7.38**	1.992	86.45
**Recovery index (RI)**	9.04	57.82	48.27	9.37	9.98**	10.655	89.98
**Drought tolerance index (DTI)**	2.7	7.03	5.62	0.75	10.76**	0.891	90.71

**Significant at the 0.01 level of the probability.

**FIGURE 1:**
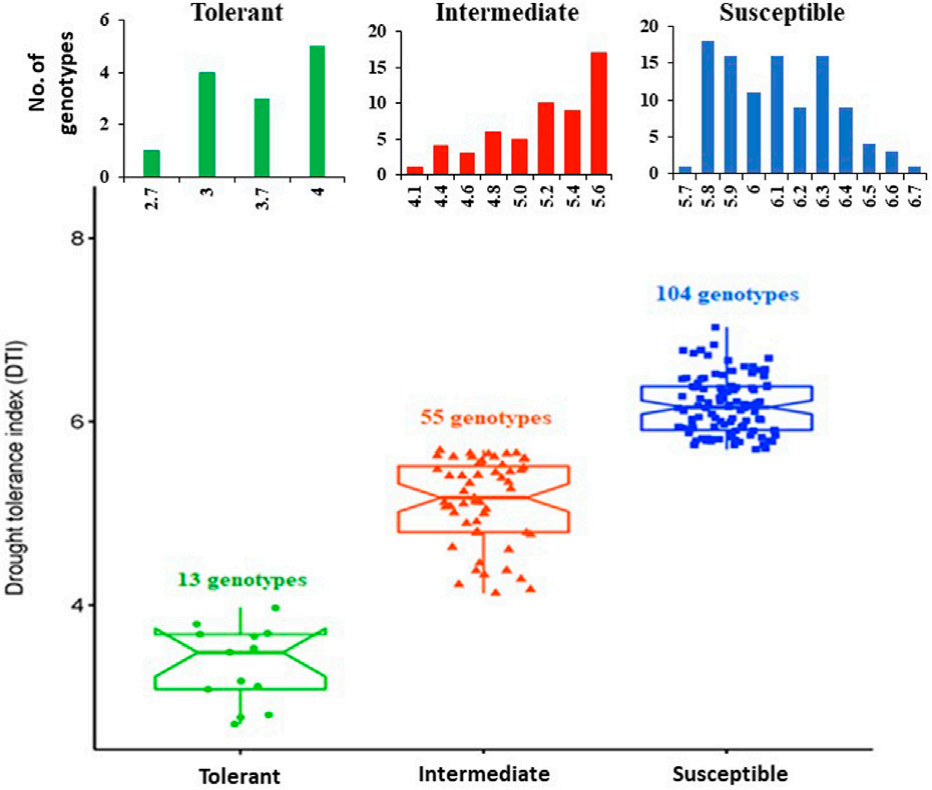
The phenotypic variation among genotypes in drought tolerance index (DTI).

The distribution of all genotypes in relation to all traits scored under drought stress is presented in Supplementary Figure S5A,B. Three drought indices were calculated to better describe drought tolerance in wheat. The phenotypic variation among the genotypes for three drought indices (RI, TI, and DTI) is illustrated in Figure 1 and Supplementary Figure S5C. The drought tolerance index (DTI) divided the genotypes into three categories: tolerant (13 genotypes), intermediate (55 genotypes), and susceptible (104 genotypes) (Figure 1). Based on the DTI, the Egyptian cultivar Shandweel-1 (DTI = 2.7) was identified as the most drought tolerant, while the United Kingdom cultivar Little Tich was the most susceptible (DTI = 6.6).

#### Genetic variation in the physiological traits

The physiological traits were scored under drought and control conditions, and the ANOVA showed highly significant differences among all genotypes (Table 3 Supplementary Table S3). The ANOVA analysis also showed highly significant differences between treatments (control vs. drought). The differences among the three biological replicates were insignificant except for the protein and proline contents. The interaction between genotypes and treatments was highly significant. The *F-values* among the genotypes for all of the physiological traits were higher under drought stress when compared to the control (Table 4 Supplementary Table S4), except for those of fructose content. All physiological traits showed very high heritability estimates in both conditions, as the heritability estimates under drought were higher than in the control condition except the fructose trait had an H^2^ of 97.42 and 96.98 under control and drought, respectively. Under control conditions, the heritability varied from 96.41 for Protein to 98.68 for proline, while under drought stress the heritability ranged from 96.98 for Fructose to 99.9 for proline (Table 4).

**TABLE 3:** Analysis of variance (ANOVA) for the physiological traits scored under control and drought conditions.

Source of variance	d.f	Protein	Amino acid	Proline	Glucose	Fructose	Total soluble carbohydrate
**Treatments (T)**	1	291.03**	53.41**	14.61**	2279.08**	19.46**	914.61**
**Replications (R)**	2	7.43**	0.66	3.57*	0.1	0.97	1.17
**Genotypes (G)**	171	25.76**	59.32**	30.89**	149.28**	44.65**	96.34**
**T x G**	171	38.14**	69.17**	87.53**	168.36**	34.08**	86.75**

*, **stander for significant levels *p* ≤ 0.05 and *p* ≤ 0.01, respectively.

**TABLE 4:** Descriptive statistics and F-values among genotypes for all physiological traits scored under control and drought conditions.

Trait	Min.	Max.	Mean	LSD	*F-value*	SD	H^2^
Control							
**Proteins (P)**	89.16	344.74	140.68	18.74	27.88**	35.55	96.41
**Amino acids (AM)**	1.85	22.94	8.74	1.61	46.72**	3.96	97.86
**Proline (PRO)**	0.76	3.55	1.63	0.15	75.78**	0.47	98.68
**Glucose (G)**	37.14	145.78	73.33	6.54	62.02**	18.51	98.39
**Fructose (F)**	22.50	261.43	86.69	15.78	38.76**	35.31	97.42
**Total soluble Carbohydrate (TSC)**	136.43	331.10	202.55	19.49	32.29**	39.78	96.9
**Drought**							
**Protein (P)**	27.57	253.78	127.74	17.89	34.87**	37.98	97.13
**Amino acids (AM)**	0.34	29.59	9.09	1.40	89.41**	4.74	98.88
**Proline (PRO)**	0.15	16.63	2.45	0.08	871.59**	2.72	99.99
**Glucose (G)**	20.73	209.08	85.47	4.79	342.77**	31.87	99.71
**Fructose (F)**	13.57	238.99	88.17	20.97	33.12**	43.38	96.98
**Total soluble Carbohydrate (TSC)**	113.57	476.93	226.73	24.70	118.71**	96.73	99.16

**Significant at the 0.01 level of the probability.

**FIGURE 2:**
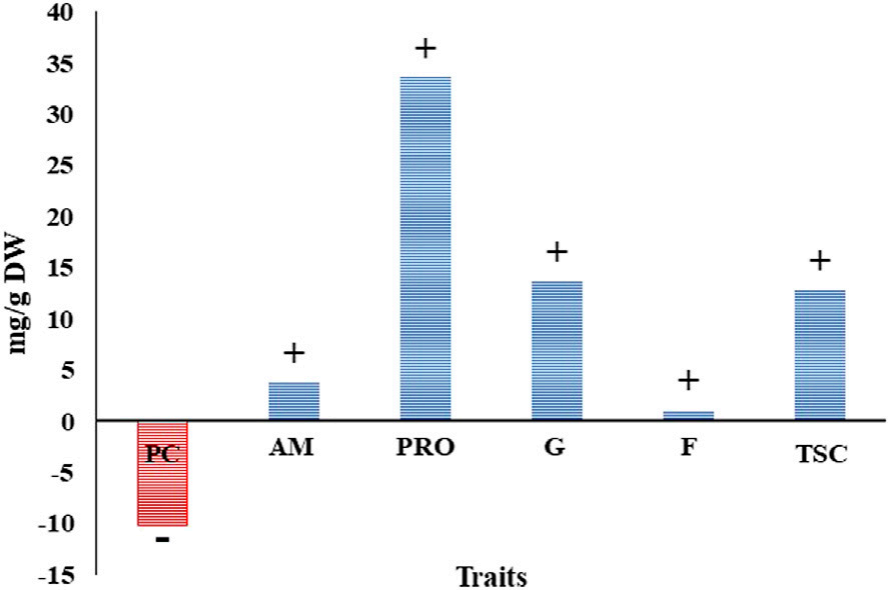
Changes in physiological traits of all tested genotypes (%) due to frought stress at the seedling stage.

Generally, the averages for all the physiological traits under drought stress were higher than those under well-watered conditions (Control), except in protein content (Table 4 and Supplementary Figure S6). The physiological changes in the leaves (reduction or increase) due to drought stress are illustrated in Figure 2. On average, all physiological traits increased due to drought stress, ranged from 1.07% (F) to 33.6% (Pro), except the protein content, which had a reduction of 10.12%. Amino acid, total soluble carbohydrate, and glucose increased by 3.8, 12.8, and 13.68 mg/g DW, respectively.

### Phenotypic and genotypic correlations

#### Correlations among the morphological traits

The phenotypic and genotypic correlations among all traits are presented in Table 5. The genotypic correlations among the traits were higher than those of the phenotypic correlations. The phenotypic and genotypic correlations among the recovery traits were higher than the correlations among the tolerance traits. SL, which was scored before exposing the plants to drought, was found to be highly and significantly associated with all traits scored in this study. For the tolerance traits, SL was found be positively correlated with S_LW (r = 0.50**) and negatively correlated with DTW (r = −0.68**). The SL showed a significant correlation with the recovery traits as it was positively correlated with DTR (r = 0.51**) and negatively correlated with both RB (r = −0.55**) and DSR (r = −0.56**). Moreover, SL had a positive and significant correlation with three selection indices.

**TABLE 5:** Phenotypic (**bold font**) and genotypic (normal font) correlations among all traits scored in this study.

Traits	Seedling traits	Tolerance traits		Recovery traits			Drought indices		
	SL	S_LW	DWT	DTR	RB	DSR	TI	RI	DTI
**SL**	1	0.50**	-0.68**	0.51**	-0.55**	-0.56**	0.55**	0.54**	0.62**
**S_LW**	0.53++	1	-0.79**	0.57**	-0.54**	-0.57**	0.99**	0.58**	0.88**
**DTW**	-0.74++	-0.86++	1	-0.49**	0.49**	0.50**	-0.85**	-0.50**	-0.76**
**DTR**	0.55++	0.63++	-0.55++	1	-0.87**	-0.95**	0.58**	0.99**	0.88**
**RB**	-0.59++	-0.60++	0.54++	-0.92++	1	0.90**	-0.56**	-0.93**	-0.83**
**DSR**	-0.60++	-0.62++	0.56++	-1.00++	0.95++	1	-0.58**	-0.97**	-0.87**
**TI**	0.59++	1.00++	-0.90++	0.63++	-0.61++	-0.63++	1	0.59**	0.89**
**RI**	0.58++	0.63++	-0.56++	0.99++	-0.95++	-1.00++	0.63++	1	0.89**
**DTI**	0.66++	0.90++	-0.80++	0.90++	-0.87++	-0.90++	0.90++	0.91++	1

*, **Significant at the 0.05 and 0.01 level of the probability, respectively. +, ++Coefficient is larger than one time and two times the standard error, respectively.

For tolerance traits, the sum of leaf wilting (S_LW) was negatively phenotypic and genotypic correlated with DTW r _phenotypic_ (p) = -0.79**, *p* < 0.01, (r _genotypic_ (g) = -0.86++). Tolerance traits had a high significant correlation with all three selection indices.

For recovery traits, on the other hand, highly significant phenotypic and genotypic correlations were found among the recovery traits scored after irrigating the drought-stressed plants. Days to regrowth had negative phenotypic (r_p_) and genotypic correlations (r_g_) with RB (r_p_ = −0.87**, r_g_ = −0.92++) and DSR (r_p_ = −0.95**, r_g_ = −1.00++). Regrowth biomass had high phenotypic and genotypic correlations with DSR (r_p_ = 0.90**, r_g_ = 0.95++). The recovery traits also had highly significant correlations with the three selection indices.

By looking at the phenotypic and genotypic correlations between the tolerance and recovery traits, it was observed that there was a significant correlation between the two groups of traits (Table 5). Sum of leaf wilting (S_LW) was negatively correlated with RB and DSR but positively correlated with DTR. Days to wilting (DTW) had a lower significant phenotypic and genotypic correlation size with the recovery traits compared to SLW. Sum of leaf wilting (S_LW) was found to have the same phenotypic correlation with DTR (r_p_ = 0.57**) and DSR (r_p_ = −0.57**), while DTW had the same correlation with DTR (r _p_ = −0.49**) and RB (r _p_ = 0.49**).

The tolerance index (including S_LW, DTW, and SL) had a higher significant phenotypic and genotypic correlation with tolerance traits than recovery traits. Likewise, the recovery (including DTR, RB, and DSR) index was found to have highly significant phenotypic and genotypic correlations with the recovery traits compared with the tolerance traits. The recovery index was positively and significantly correlated with the tolerance index, with a correlation value of 0.59**. Interestingly, DTI (including TI and RI) was highly and significantly correlated with all traits (tolerance and recovery traits) assessed in this study. The highest correlation between DTI and the tolerance traits was for S_LW (r = 0.88**), while DTR, among the recovery traits, had the highest correlation with DTI (r = 0.88**). The DTI had the same significant phenotypic correlation with RI and TI (r _phenotypic_ = 0.89**).

#### Correlation between the physiological and morphological traits under drought stress

The correlation coefficients between all morphological and physiological traits under drought stress are shown in Table 6. The physiological, protein (P), proline (PRO), and fructose (F) did not have any significant correlations with any of the morphological traits. Amino acid (AM) had positive and significant correlations with S-LW (r = 0.27**), SL (r = 0.24**), and DTR (r = 0.36**). Furthermore, AM had negative correlations with DTW (r = −0.26**), RB (r = −0.38**), and DSR (r = −0.39**). A positive and significant correlation was found between AM and the three selection indices (TI, RI, and DTI). Notably, both G and TSC had similar association trends with the morphological traits, as they had negative and significant correlations with S_LW, SL, and DTR (Table 6) and positive significant correlations with DTW, RB, and DSR. The selection indices had negative and significant correlations with G and TSC.

**TABLE 6:** Correlations between morphological and physiological traits under drought stress at the seedling stage.

	Physiological traits						
Morphological traits	Traits	P	AM	PRO	G	F	TSC
**SL**	-0.067	0.237**	0.052	-0.211**	-0.003	-0.278**
**S_LW**	-0.061	0.274**	0.03	-0.186*	0.03	-0.168*
**DTW**	0.025	-0.261**	0.008	0.157*	0.019	0.163*
**DTR**	-0.108	0.361**	-0.062	-0.322**	-0.077	-0.367**
**RB**	0.097	-0.376**	0.013	0.251**	0.006	0.271**
**DSR**	0.071	-0.388**	0.094	0.288**	0.114	0.392**
**TI**	-0.058	0.282**	0.026	-0.190*	0.024	-0.176*
**RI**	-0.105	0.374**	-0.054	-0.310**	-0.066	-0.354**
**DTI**	-0.092	0.367**	-0.016	-0.280**	-0.025	-0.297**

*, ** significant at the 0.05 and 0.01 level of the probability, respectively.

### Phenotypic selection

#### Morphological traits

To select the most promising genotypes with high drought tolerance from both the tolerance and recovery traits at the seedling stage, the genotypes were sorted for all traits based on the direction of drought tolerance from most tolerant to susceptible. Then, the 20 most tolerant genotypes in each trait were selected. Finally, genotype was selected from the top 20 genotypes if it was tolerance criteria in at least five traits and DTI. A set of 13 genotypes were ultimately identified as drought tolerant (Supplementary Table S1). Six of these 13 tolerant genotypes (MISR1, SAKHA93, Shandweel-1, PI525434, SIDS13, and Hutch) were among the 13 most drought-tolerant genotypes for nine traits, while three (SIDS12, Gimmeiza-12, and Sohag-3) were among the 13 most drought-tolerant genotypes in eight traits. The genotypes Beni Swief-5 and Beni Swief-7 were tolerant to drought for seven and six traits, respectively (Table 7). The ramming two genotypes (Gimmeiza 11 and Gimmeiza-07) were tolerant to drought in five traits. All 13 genotypes had a short height, high days to wiling, regrowth biomass after drought, and high survival rate after drought, and low levels of wilt. Finally, based on the number of traits and the value of DTI, the 10 most drought-tolerant genotypes were selected, and their physiological changes to drought and specific drought genes were investigated (Table 7).

**TABLE 7:** The most promising spring wheat genotypes with high drought tolerance and the 10 most drought susceptible genotypes at the seedling stage.

Genotype	country	DTI^V^	N. of traits	SL	S_LW	DTW	DTR	DSR	RB	TI	RI	DTI
**The most tolerant genotypes**												
**Shandweel-1**	**Egypt**	**2.7**	**9**	+	+	+	+	+	+	+	+	+
**SIDS13**	**Egypt**	**2.77**	**9**	+	+	+	+	+	+	+	+	+
**MISR1**	**Egypt**	**2.8**	**9**	+	+	+	+	+	+	+	+	+
**SAKHA93**	**Egypt**	**3.17**	**9**	+	+	+	+	+	+	+	+	+
**PI525434**	**Morocco**	**3.48**	**9**	+	+	+	+	+	+	+	+	+
**Hutch**	**United States**	**3.66**	**9**	+	+	+	+	+	+	+	+	+
**SIDS12**	**Egypt**	**3.08**	**8**	+	+	+	+	+		+	+	+
**Sohag-3**	**Egypt**	**3.53**	**8**	+	+		+	+	+	+	+	+
**Gimmeiza-12**	**Egypt**	**3.68**	**8**		+	+	+	+	+	+	+	+
**Beni Swief-5**	**Egypt**	**3.11**	**7**			+	+	+	+	+	+	+
Beni Swief-7	Egypt	3.97	6	+			+	+	+		+	+
GIMMIZA11	Egypt	3.69	5	+	+				+	+		+
Gimmeiza-07	Egypt	3.79	5	+	+				+	+		+
**The most susceptible genotypes**												
Little Tich	United Kingdom	7.03	8		#	#	#	#	#	#	#	#
Rhodesian Sabanero	Kenya	6.78	7		#	#	#	#		#	#	#
PI238391	Kenya	6.75	7		#		#	#	#	#	#	#
Hmira	Tunisia	6.67	7		#		#	#	#	#	#	#
Grekum 105	Kazakhstan	6.72	6		#		#	#		#	#	#
Kenya Governor	Kenya	6.51	6	#			#	#	#		#	#
PI525221	Morocco	6.84	5		#			#	#	#		#
Atson	United Kingdom	6.51	5				#	#	#		#	#
PI525318	Morocco	6.2	5	#			#		#		#	#
Musane	Oman	6.56	4	#	#					#		#

+Refers that the genotype was among the highest performance genotypes in the trait, while, # refers that the genotype was among the lowest performance genotypes in the trait.

**DTI** ^**V**^, refers to the values of drought tolerance index (DTI) for each genotype.

**The bold** font indicates the most 10 drought-tolerant genotypes were selected and used to study the physiological changes and genetic analysis.

#### Important physiological changes among tolerant and susceptible genotypes

In addition to the 10 most drought tolerant genotypes at the seedling stage, the 10 most susceptible genotypes were also selected to give a reliable image of the main differences between the tolerance and susceptible genotypes (Table 7).

**FIGURE 3:**
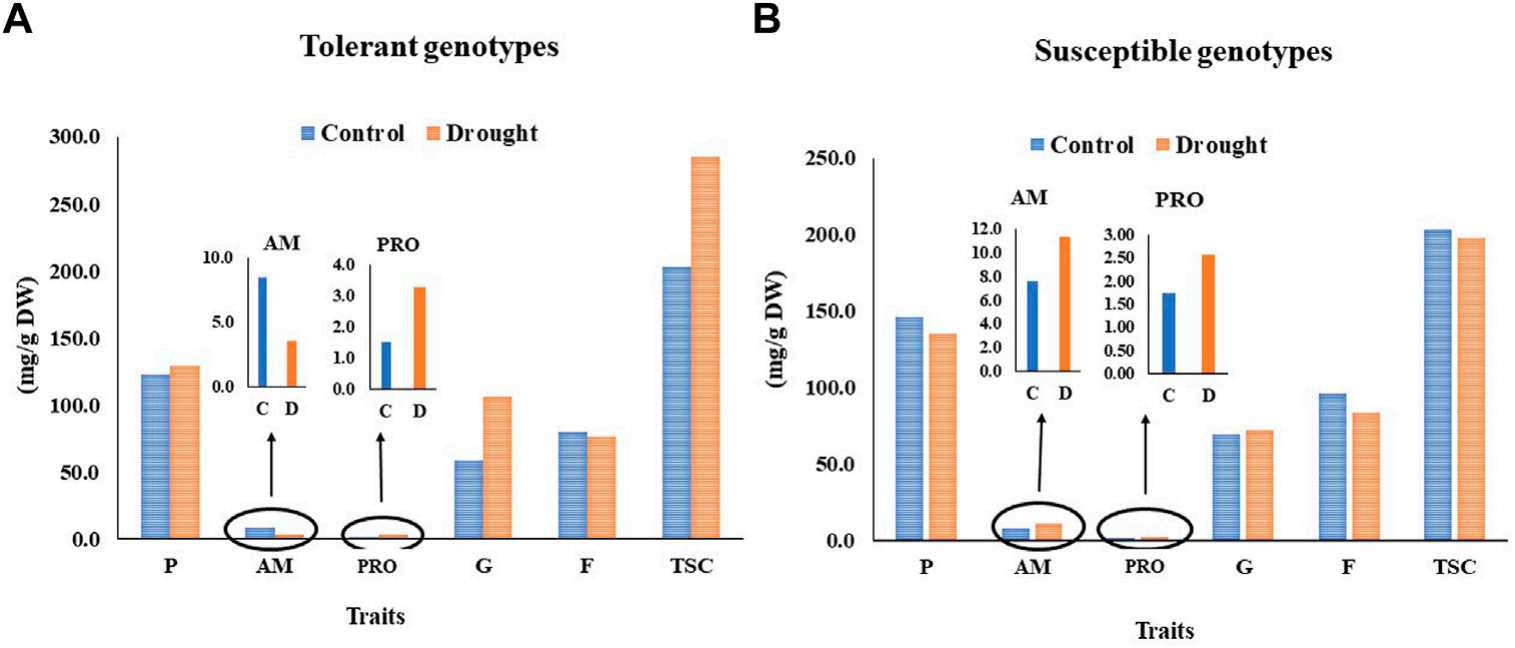
The variation between the most tolerant **(A)** and susceptible **(B)** genotypes in the content of the physiological traits under control nd drought stress.

Comparisons between the 10 most drought tolerant and susceptible genotypes (Figures 3A,B), showed that the drought-tolerant genotypes had increased G, TSC, P, and PRO traits, but decreased F and AM traits under drought stress (Figure 3A). For the tolerant genotypes, the soluble proteins, proline content, glucose, and total soluble carbohydrate content increased under drought stress compared to the control. In contrast, F and AM decreased under drought stress compared to the control, whereas PRO increased by 53% compared to control. While F and AM were decreased by drought stress competed with the control conditions (Figure 3A). The susceptible genotypes also had increased PRO, AM, and G under drought stress, while there was a notable decrease in TSC, F, and P compared to the control (Figure 3B). The highest increase in AM was identified in the susceptible group.

### Genetic analyses of the selected genotypes

#### Screening of the tolerant genotypes for drought *DREB*-specific genes

**FIGURE 4:**
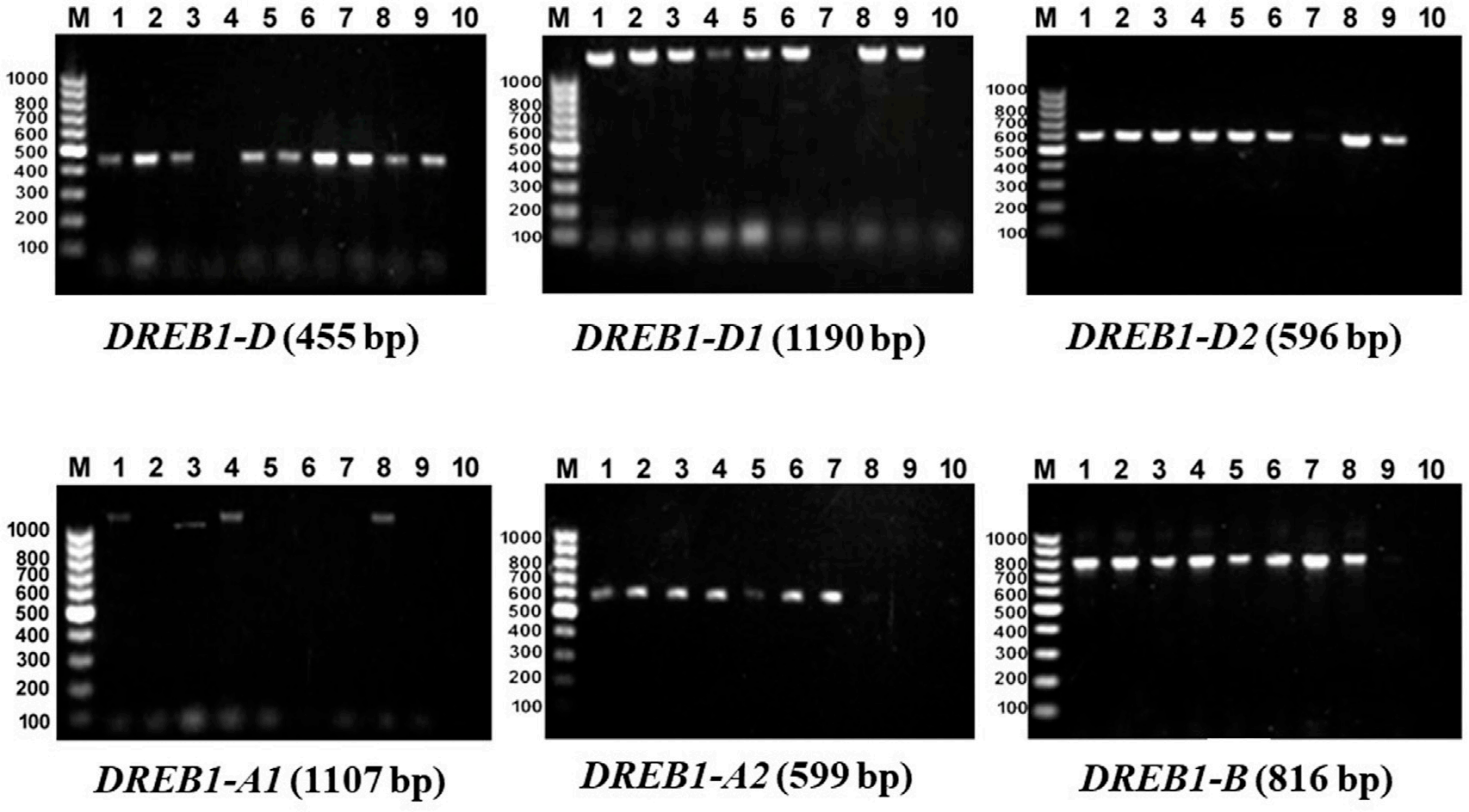
Agarose gel electrophoresis of DREB genes used in this study. Names of genotype are mentioned in Table 8.

The 10 most drought-tolerant genotypes (Table 7) in this study were tested for the presence of nine specific *DREB* alleles. The genotyping revealed the amplification of only six *DREB* genes, as three primers did not give any specific bands (Figure 4). For the six amplified primers, the presence/absence of each gene was scored in each genotype (Table 8). The six primers showed clear polymorphism among the 10 most tolerant genotypes. MISR1 and SAKHA93 had six different *DREB* genes, while the American genotype Huch had one (*DREB1*-D1). The SIDS12, Gimmeiza-12, Shandweel-1, Beni Swief-5, and SIDS13 genotypes each contained five genes, while the Sohag-3 and PI525434 genotypes contained three.

**TABLE 8:** The total number of *DREB* genes present in each of the ten drought-tolerant genotypes.

No. of* genotypes	Genotype*	Country	*DREB1 -A1*	*DREB1 -A2*	*DREB1 -B*	*DREB1 -D*	*DREB1 -D1*	*DREB1 -D2*	DTI^V^	Total
1	MISR1	Egypt	+	+	+	+	+	+	2.8	6
2	SIDS12	Egypt	-	+	+	+	+	+	3.17	5
3	SAKHA93	Egypt	+	+	+	+	+	+	3.11	6
4	Gimmeiza-12	Egypt	+	+	+	-	+	+	3.68	5
5	Shandweel-1	Egypt	-	+	+	+	+	+	2.7	5
6	Beni Swief-5	Egypt	-	+	+	+	+	+	3.08	5
7	Sohag-3	Egypt	-	+	+	+	-	-	2.77	3
8	SIDS13	Egypt	+	-	+	+	+	+	3.48	5
9	PI525434	Morocco	-	-	-	+	+	+	3.53	3
10	Hutch	United States	-	-	-	+	-	-	3.66	1

Where, the positive sign (+) indicates the presence of the gene, while the negative sign (-) indicates the absence of the gene or indicates that the genotype does not contain this gene. * Indicates the positions and names of genotypes in the gel during the electrophoresis. **DTI** ^**V**^, refers to the values of drought tolerance index (DTI) for each genotype.

#### Genetic distance and dendrogram analyses of the tolerant genotypes

**FIGURE 5:**
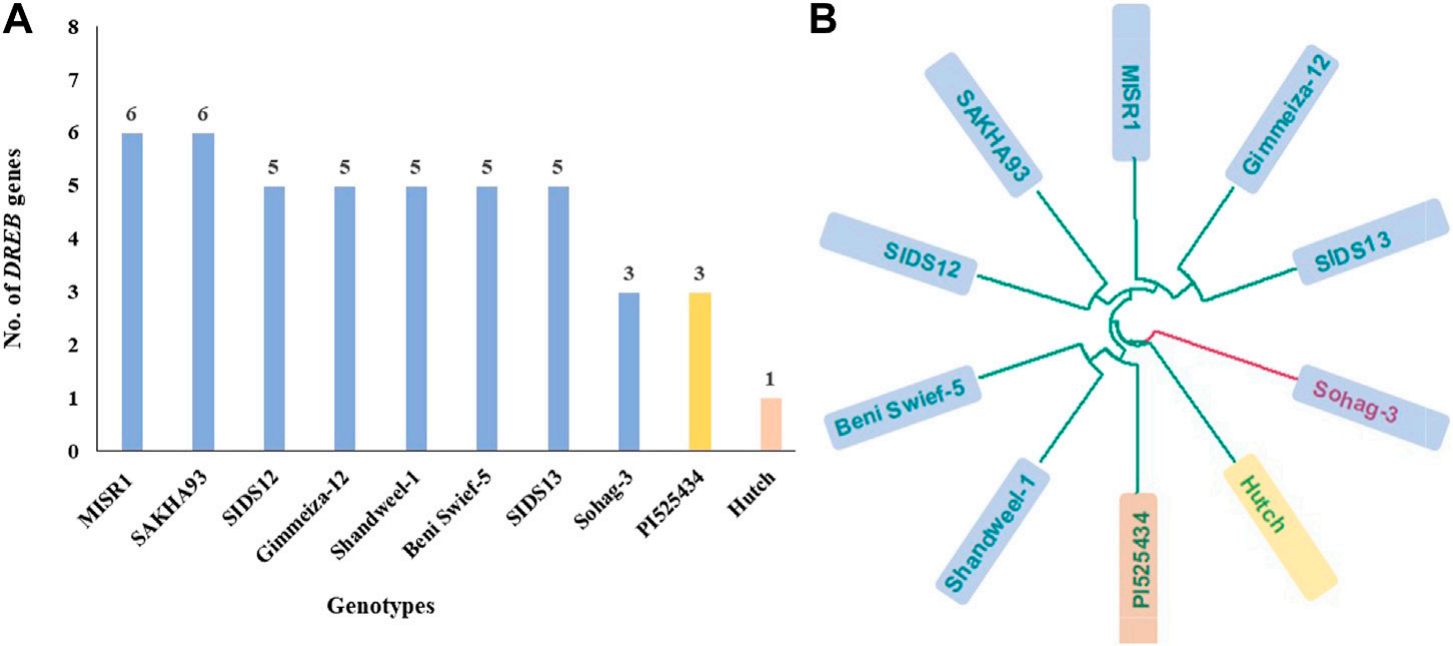
**(A)** The total number of DREB genes present in each of the ten tested genotypes. **(B)** Dendrograms analysis showing the relationship among the most ten drought-tolerance genotypes based distance, blue color refer to egyptian genotypes, yellow color refer to Moroccan genotyes and red color refer to American genotype.

The genetic distance among the most tolerant genotypes was calculated using 21,450 SNP markers (Figures 5A,B, and Supplementary Table S5). The analysis of the dendrogram divided the tolerant genotypes into three different branches. The branch I included eight Egyptian genotypes and one from Morocco. Branches II and III each included one genotype, Sohag 3 (Egypt) and Hutch (USA), respectively. The genetic distances among the genotypes ranged from 0.189 (Gimmeiza-12 and SIDS13) to 0.488 (MISR1 and Sohag-3). Sohag-3 had a genetic distance of >0.46, and Hutch had a genetic distance of >0.39 when compared with all genotypes.

## Discussion

### Genetic variation in morpho-physiological traits associated with drought tolerance at the seedling stage

#### Morphological traits

The high significant genetic variation among the genotypes in all traits was believed to be due to the diversity among these genotypes, as they were from 20 different countries covering all continents (except Antarctica). The germplasm used in this study could be utilized to detect a large amount of genetic variation related to drought tolerance in wheat at the seedling stage and could thus be fruitful for plant breeders to help discriminate between tolerant and susceptible genotypes.

Two types of drought tolerance trait, tolerance, and recovery, were scored in this investigation, and the data provided will help to identify the different mechanisms of drought tolerance in wheat at the seedling stage (Sallam et al., 2018b; Sallam et al., 2022).

Tolerance traits reflect the ability of a plant to tolerate prolonged drought stress. Leaf wilting and days to wilting are tolerance traits which directly associated with drought tolerance (Bowne et al., 2012; Muir and Thomas-Huebner, 2015; Drira et al., 2016; Sallam et al., 2019). Leaf wilting indicates a deficiency in soil moisture and subsequent water uptake and transport to the shoots (Sanad et al., 2016). To better reflect the symptoms of water deficiency on the leaves, all five scores (LW1, LW2, LW3, LW4, and LW5) were summed up. SLW is an important accumulative trait that reflects the effects of drought stress on plant leaves. Many previous studies only scored leaf wilting once during the drought treatment (Sayed et al., 2012; Pathan et al., 2014). However, scoring leaf wilting multiple times during the drought treatment enables the precise evaluation of drought tolerance for each respective genotype over time (Sallam et al., 2019). In this investigation, both traits showed large variation among the genotypes, ranging from 14 to 24 and 4–9 days for SLW and DTW, respectively.

In contrast, recovery traits describe a plant’s ability to regrow and recover after irrigation following prolonged drought stress. Bearing in mind that these traits were scored after cutting and reirrigating the drought-stressed plants. Therefore, these traits not only tested the drought tolerance in plants but also reflected their ability to produce new shoots after drought stress. These essential traits should be considered together when scoring plants after irrigation. It was noted that some genotypes started to regrow only a few days after irrigation, such as Gimmeiza-07 and SIDS12, but at the end of the experiment, these genotypes had very little regrowth. Therefore, selection should be made carefully for genotypes that regrow after a few days but also have high regrowth biomass at the end of the drought experiment. Regrowth in wheat after drought conditions has been scored at the seedling stage. Previous studies, such as Pearce (1985), scored the regrowth of wheat seedlings after drought by measuring leaf height before and after rewatering within a few hours and on subsequent days.

The results from this investigation indicate that three selection indices (TI, RI, and DTI) should be utilized to select the most drought-tolerant genotypes. The drought-tolerant index was the main index, as it included both indices. The most important feature of the selection index developed by Falconer (1996) is the possibility of including more than one target trait. In this investigation, high levels of variation were found among all genotypes based on the three selection indices, which could thus be utilized to select promising drought-tolerant genotypes.

The high H^2^ levels found for all traits scored in this study promise effective selection by which to improve drought tolerance in wheat at the seedling stage. SLW and DTW had H^2^ values of 0.84 and 0.83, respectively. Sallam et al. (2018b) reported approximate heritability for SLW with an H^2^ of 0.82, while their DTW value for H^2^ was 0.72, which was lower than that reported in this study. High heritability estimates were also identified for the recovery traits (0.84–0.91), and they were higher than those reported by Sallam et al. (2018b) (0.74–0.88). Likewise, the heritability of the three selection indices reported in this study was also higher than those reported by Sallam et al. (2018b). These results differed due to the nature of the populations used in the two studies. The diverse population of this study possessed higher genetic diversity and variation when compared with the biparental population tested by Sallam et al. (2018b) for drought tolerance. Recovery and tolerance traits were recently reported under drought stress by Sallam et al. (2022) in a diverse winter wheat population at the seedling stage. They reported that the recovery traits had higher H^2^ (0.75–0.89) estimates than the tolerance traits (0.71–0.84). These results further support the H^2^ results obtained in this study on spring wheat.

#### Selection of the most promising drought-tolerant genotypes

As described, high levels of genetic variation and heritability estimates allow for selecting the most drought-tolerant genotypes for further improvement through plant breeding programs. Most earlier studies depended on single trait selection to improve tolerance to stress which is not recommendable (Sallam et al., 2019). Instead, multiple trait selection is more fruitful when selecting the target genotypes.

The 13 genotypes selected were from Egypt (11), the USA 1) and one genotypes was from Morocco country. The tolerant genotypes had diversity in the traits correlated to their tolerance. Identifying different genetic resources to investigate drought tolerance will undoubtedly help to improve this trait in spring wheat at the seedling stage and expand the circle of genetic diversity in Egypt. Thus, genotypes could be selected from this set and used as candidate parents for crossing into future breeding programs to improve drought tolerance. Of the 13 genotypes, Shndweel-1, PI525434, Sakha 93, Hutch, Sids 13, Misr 1, Sids 12, Sohag 3, Gimmieza 12, and Beni Swief 5, were selected as the ten most drought tolerant for both the tolerance and recovery traits. They could thus be used in future breeding programs to accelerate the improvement of drought tolerance in wheat. These ten highly drought-tolerant genotypes had the lowest DTI (Table 7). The DTI provides a valuable method by which to truly select the most drought-tolerant genotypes with superior performance in more than one trait. These ten genotypes were used for further physiological and genetic studies.

#### Physiological analyses under drought stress

In this study, the physiological changes at the seedling stage were evaluated by assessing six physiological traits (protein, amino acids, proline, glucose, fructose, and total soluble carbohydrates) to understand the biochemical changes that occur in wheat leaves in response to drought stress and consequently, how to alleviate this stress. All these physiological traits had a direct relation to drought stress, and some previous studies, such as Abid et al. (2018), used these traits to study this trait at the tillering and jointing stages, respectively.

These high levels of genetic variation among genotypes in all physiological parameters could provide an extremely useful resource for both breeders and geneticists to efficiently understand the changes in the physiological parameters that occur in plants to alleviate the effects of drought stress. Moreover, the high levels of genetic variation indicated that there was also variation in the ability of the different genotypes to make substantial changes to their physiological parameters. The presence of significant differences between the two different treatments indicated that drought affects the performance of the genotypes when compared to their performance under the control conditions. Previous studies, such as Nowsherwan et al. (2018), stated that the performance of the genotype varies according to the different conditions and stages. Moreover, it was observed that the genotype responses differed with the different treatments, and this was shown by the presence of significant variation between the G × T interactions in all physiological traits. This effect can be observed by estimating the changes in the physiological parameters due to drought stress in the genotypes (Figure 2). The most significant change was in the proline content, which increased by 33.6% under drought stress, and its accumulation was reported in stressed plants compared to non-stressed plants (Sallam et al., 2018a; Dawood et al., 2020). In the current study, the physiological traits all had high H^2^ estimates under both control and drought conditions, but they were higher under drought conditions when compared with the control. These higher heritability estimates (H^2^) suggested that using physiological traits to improve drought tolerance would be more successful.

On average, for each population in this investigation, the physiological traits increased under drought stress compared to the control, except for protein content (Table 4 and Supplementary Figure S6). This indicates that the genotypes use different mechanisms by which to deal with and adapt to drought stress fluctuations. This result was expected due to the high levels of genetic diversity in the tested genotypes, which were collected from 20 different countries. In addition, studying the physiological changes that occur because of drought stress was useful as they provided information on the different ways in which plants function to counteract and relieve stress. The study of these changes showed that the content of all physiological traits increased or accumulated in the leaves except protein content. The soluble protein content decreased by 10.12%, indicating that the protein content was the trait most affected by drought stress. It is clear in this study that the protein content was decreased in favor of the proline and amino acid increases (Figure 2). Gilbert et al. (1998) reported that the protein reduction could be associated with an increase in amino acids that may serve as a readily available energy source or as a nitrogen source during limited growth and photosynthesis and the detoxification of excess ammonia under periods of stress.

Physiological and developmental plant responses to drought were shown to occur by reprogramming gene expression and metabolism (Reddy et al., 2004; Chaves et al., 2009; Hayano-Kanashiro et al., 2009; Zhang et al., 2014). Responses to drought stress depend on plant species, the stage of development, the rate of dehydration, and the duration and severity of stress (Reddy et al., 2004; Chaves et al., 2009). To elucidate a plant’s ability to survive under drought conditions, it is important to study the physiological, biochemical, and genetic basis of adaptation and tolerance as well as the mechanisms of recovery under drought stress. The physiological modifications in response to water stress were studied, herein, in terms of soluble proteins, amino acids, proline, glucose, fructose, and total soluble carbohydrate contents, which were analyzed under drought and control conditions to understand the biochemical changes in shoot metabolites in spring wheat plants. The average amount of glucose, fructose, and total soluble sugars (as the average of 172 cultivars or genotypes) increased in response to water stress, but for fructose, the increase was very slight. The accumulation of sugars in response to drought stress was not uniform, indicating that there were categorical tasks in charge of each sugar component. Fructose only slightly responded to the drought stress.

### Correlation analyses

#### Correlations among morphological traits

The phenotypic and genetic correlations among all traits scored in this study shed light on the different drought tolerance mechanisms. Overall, the genetic correlations among the traits were higher than the phenotypic correlations, and these results corresponded with those obtained by Sallam et al. (2018b). Genetic correlation is an informative analysis as it provides predictions for the selection response in one or more traits to be made when selection is another trait (Hill, 2013).

The high level of genetic and phenotypic correlations among all traits promises fruit selection for a group of traits that are directly associated with drought tolerance. It was noted that the tolerance and recovery traits had very high and significant correlations within each group. While there was also a significant correlation between the tolerance and recovery traits, the correlation size was smaller than that within each group. The correlation between RI and TI was higher than the correlation between the traits in the two groups. This indicates that including more than one trait in a selection index could be more beneficial for selecting target traits. Interestingly, DTI had high and significant levels of correlation with all traits in both groups. These results highlighted the importance of using a selection index to improve target traits and optimize the selection to improve drought tolerance. The phenotypic correlations reported by Sallam et al. (2018b)showed that there were no correlations between the tolerance and recovery traits. Even after creating a selection index for each group, RI and TI showed no significant correlation, suggesting that different genetic mechanisms controlled these traits. The absence of correlation between the tolerant and recovery traits was also validated in a highly diverse winter wheat population by Sallam et al. (2022), conducting QTL mapping (winter biparental population) and GWAS (diverse winter wheat population). The study’s results revealed that each group of traits was controlled by different QTLs and concluded that both types of traits were controlled by different genetic mechanism different genetic mechanisms. The gene and SNP networks analysis supported this notion (Sallam et al., 2022). It should also be considered that Sallam et al. (2018b) tested winter genotypes which are completely different from the spring wheat genotypes used in this study. Winter wheat, for example, has a crop coefficient of 0.7 for nonfrozen soil, while the spring wheat crop coefficient is 0.3. The lower the crop coefficient, the lower the water demand and water stress (Allen and Zaplachinski, 1994), consequently, spring wheat is more tolerant than winter wheat. The relationship among these traits expanded our knowledge and understanding of the drought tolerance mechanisms in both spring and winter wheat; both appeared to possess different genetic mechanisms by which to alleviate the effects of drought stress. This information will be valuable for wheat breeders and geneticists as it can be utilized for the selection of drought tolerance in spring and winter wheat.

It was of note that seedling height had a significant correlation with tolerance and recovery traits in this investigation. This indicates that shorter plants were more drought tolerant. This trait could thus be used to predict and select drought tolerance as this trait was scored before exposing plants to drought stress. The same trait did not correlate with recovery and tolerance traits in winter wheat (Sallam et al., 2022).

#### Correlations between morphological and physiological traits

The correlation between morphological traits (tolerance and recovery) and physiological traits helps elucidate the changes in wheat plants at the seedling stage to alter and alleviate the effects of drought stress. Fructose was previously reported to be associated with secondary metabolite synthesis and not osmoprotectants (Younes et al., 2019). In this investigation, fructose was found to only slightly respond to drought stress conditions. It was thus determined that fructose was not involved in osmotic adjustments, which was also indicated by the correlation data, as it did not correlate with any of the studied traits for wilting or recovery. Younes et al. (2019) demonstrated that fructose might be related to the synthesis of substrates related to phenolic compound synthesis. Glucose, in contrast, which acts as a substrate for cellular respiration or osmolytes to maintain cellular homeostasis, was found to accumulate in the shoots of drought-treated plants, implying that glucose is associated with osmotic adjustments under drought stress conditions, as was reported by Misra and Gupta (2005). It is of note that glucose was found to be negatively associated with witling traits. Thus, a positive relationship was found between days to wilting and glucose content indicating that the tolerant genotypes accumulated high levels of glucose to tolerate a prolonged period of drought. The significant correlations between glucose and the recovery traits indicated that increased glucose accumulation levels improved survival and recovery after reirrigation. This result provides a clear explanation of the importance of glucose as an osmotic against drought stress. The above-described tendencies for glucose were observed for the total soluble carbohydrates, which contributed to the osmotic adjustment of wheat plants exposed to prolonged drought stress. In conformity, carbohydrates can contribute to 30–50% of the osmotic adjustment in glycophyte plants (Al-Thani and Yasseen, 2018).

The nitrogenous components of the stressed shoots showed that the soluble protein content was reduced in favor of the accumulation of free AM and proline. This increase in AM and proline under stress conditions may result from the degradation of proteins, affect their synthesis, inactivate major enzymes, and destroy membranes (Fahad et al., 2017; Sallam et al., 2018a; Dawood et al., 2020). Increased levels of free amino acids and proline were identified in different plants under abiotic stress (Sadak, 2016b, 2016a; Tawfik et al., 2017; Sallam et al., 2018a). It was concluded that these compounds have an important role in enhancing the tolerance of plant cells to various abiotic stresses by increasing the osmotic pressure in the cytoplasm and increasing the relative water content, which is essential for plant growth and different metabolic processes. The average proline levels identified for the 172 tested cultivars showed no correlation with any of the studied morphological or recovery-r traits. The collection used covered a wide range of cultivars and included variants susceptible to drought stress. Of note is that most of the studied cultivars ranged from moderately tolerant to sensitive, and only 24 cultivars were tolerant. Thus, the categorization of these cultivars based on their drought responses will likely provide a reliable image of their physiological behavior in relation to the recovery and tolerance traits.

The AM was positively correlated with three drought indices (drought susceptibility). In this regard, the high levels of amino acid content may be associated with the degradation of proteins which were found to be reduced in response to drought. Thus, drought stress instigated the solubilization of proteins, which may have negative effects on the main enzymes related to various physiological processes. The production of high levels of amino acids under stress conditions could impact multiple processes. The high synthesis rates of the amino acids can result from proteolysis, or their consumption can be restricted due to a decrease in protein synthesis or secondary metabolite production (Silva et al., 2019). Araújo et al. (2011) reported that amino acids can be utilized as alternative respiratory substrates and provide stressed plants with an additional energy source during an energy deprivation situation. In situations with insufficient carbohydrate supply due to a decrease in photosynthesis rates, which usually occurs during stress conditions, plants can utilize amino acids as alternative substrates for mitochondrial respiration (Braun et al., 2015; Hildebrandt, 2018). The degradation pathways of some amino acids have been identified as essential factors for dehydration tolerance in Arabidopsis (Pires et al., 2016). Overall, the general metabolism of the studied cultivars was found to be shifted toward energy saving and stress defense, which leads to an arrest of growth and development. Plants start to invest their energy resources into the production of protective secondary metabolites and osmolytes to counteract the effects of drought stress.

To understand the physiological changes that occurred in plants to alleviate the effect of drought stress, their physiological characteristics were studied in the most drought-tolerant and susceptible genotypes (Figures 3A,B). The increase or decrease of each physiological trait because of drought stress in both the tolerant and susceptible genotypes was estimated. The increase or decrease in physiological components differed according to the stage in which the stress occurs, the duration and severity of the stress, as well as the performance of the genotypes (Reddy et al., 2004; Chaves et al., 2009). The ability of tolerant plants to respond to drought tolerance depends on the genotype. For example, the drought tolerance mechanisms of some genotypes include the accumulation of soluble sugars, proline content, amino acids, chlorophyll content, and enzymatic and nonenzymatic antioxidant activities (Abid et al., 2016). In this study, clear differences were observed between the drought-tolerant and susceptible genotypes for these physiological components, and there was no clear trend as some traits increased and others decreased. These genetic differences in the physiological components between genotypes can be used to effectively identify the genes controlling the physiological traits and accelerate the genetic improvement of drought tolerance (Bowne et al., 2012). The drought-tolerant genotypes showed apparent increases in their protein, proline, and sugar content and decreased their amino acid levels compared to the control, and the opposite occurred in the drought-susceptible genotypes (Figures 3A,B). The most important characteristic distinguishing the tolerant genotypes from the susceptible genotypes under drought stress is a significant increase in proline content, which increased by 50%, while amino acids were reduced by 65%. The drought-tolerant genotypes accumulated more proline in their leaves under drought stress at the expense of other free amino acids. In contrast, the drought-susceptible genotypes increased the amino acids in their leaves at the expense of their proline content. This indicated that the tolerant genotypes possessed genetic plasticity that allowed them to accumulate increased levels of proline under drought conditions, and this was utilized as a self-protection mechanism by which to counteract drought stress. Previous studies, such as Basudeb et al. (2019), reported that the presence of proline is a common trait in most cereals under drought stress Zali and Ehsanzadeh (2018) previously stated that only a few plant species can produce enough proline to notably reduce abiotic stress effects and thus utilize proline as another tolerance mechanism against drought stress. Moreover, proline is a source for several amino acids and nitrogenous compounds (Britikov et al., 1970). It also contributes to the stabilization of subcellular structures, the scavenging of reactive oxygen species, and the buffering of cellular redox potential under stress (Ashraf and Foolad, 2007).

### Genetics analysis for the most drought tolerant genotypes

#### Screening the tolerant genotypes for drought using *DREB*-specific genes

Integrating specific DNA molecular markers for target traits in the breeding program will help in accelerating the breeding program. In this study, nine specific primers associated with nine different allelic variants of *TaDREB1* genes developed by Liu et al. (2018) were used to screen the most drought tolerant genotypes for the presses of *DREB* genes. *DREB* genes constitute a large family and belong to transcription factors (TFs) that stimulate the expression of many functional genes (Agarwal et al., 2006; Samarah, 2016). A total of 210 *DREB* genes associated with abiotic stress tolerance (Niu et al., 2020). Identifying and validating new primers for these genes are very useful for breeding and genetics programs in wheat. Six specific *DREB* primers were polymorphic among the ten drought-tolerant genotypes (Table 8). The high polymorphism and diversity among the drought-tolerant genotypes indicated that the tolerant genotypes differed in the number of *TaDREB1* gene haplotypes. It was reported that the drought-resistant materials showed inconsistent heterogeneity of *TaDREB1* gene haplotypes and the nucleic acid polymorphisms of the *TaDREB1* gene in wheat (Yousfi et al., 2016). In the present study, it was observed that the largest number of *TaDREB1* alleles was present in both the Egyptian genotypes MISR1 and SAKHA93 (six genes), while the lowest number of these genes was present in foreigner genotypes such as Moroccan genotype PI525434 (three genes) and American genotype Hutch (one gene). The result also revealed that the Egyptian genotypes were higher drought tolerance than the Moroccan and American genotypes. By looking at the DTI of these genotypes, the correlation between number of *TaDREB1* and DTI was negative (r = -0.66**), indicating that some of high tolerant genotypes may contain other drought genes and *TaDREB1* alleles not only the source of drought tolerance in the selected genotypes. PI525434 (Morocco) and Hutch (USA) ranked fifth and sixth among the ten drought-tolerant genotypes, respectively. Therefore, they probably included other drought genes than those used in this study. These two genotypes can be utilized for crossing with the Egyptian genotypes to genetically improve drought tolerance at the seedling stage in spring wheat. Further molecular analyses should be done on these cultivars to discover more genes related to drought stress. The results of genotyping confirmed that primers of *TaDREB1* alleles used in this study were effective and valuable for marker-assisted selection to test the presence of *TaDREB1* alleles in a large number of genotypes in a short time as an alternative to breeding methods traditional.

#### Genetic distance and dendrogram analyses of the tolerant genotypes

The genetic distance analysis among the ten drought-tolerant genotypes provides valuable information on the diversity among these genotypes. Such information can be useful in selecting the candidate’s parents for future breeding programs (Eltaher et al., 2018; Mourad et al., 2020). Here, although the ten drought-tolerant genotypes included eight genotypes from Egypt, the genetic distance among them is still useful for the breeding program. Out of the ten genotypes, Sohag-3 was positioned in a separate cluster and had a genetic distance of >0.649 from all other genotypes. Unexpectedly, the two foreigner genotypes, Hutch (USA) and PI525434 (Morocco), were included in the cluster with the other seven Egyptian genotypes. Hutch had a higher range of genetic distance with the Egyptian genotypes than PI525434. The highest genetic distance was found between MISR one and Sohag-3 (0.664); however, including Hutch and PI525434 in the crosses with the Egyptian genotypes (e.g. Sohag-5) may be more fruitful for two reasons (I) increasing the genetic diversity among the Egyptian wheat gene pool and (II) the two genotypes may have other drought-tolerant genes rather than *DREBs* and the crossing with the Egyptian genotypes could produce wheat cultivars having more drought tolerance at the seedling stage. Genetic diversity is essential for plant survival in nature against the consequences of climate change and crop improvement (Bhandari et al., 2017). Including new plant genetic resources such as Hutch and PI525434 will undoubtedly provide the opportunity for wheat breeders in Egypt to develop new and improved wheat cultivars not only for drought tolerance but also for other desirable characteristics such as agronomic yield traits, quality traits, tolerance to biotic and abiotic stress tolerance, etc. (Al-khayri et al., 2019; Baenziger et al., 2021; Bhavani et al., 2021)

## Conclusion

The visual scoring of the traits used in this study is effective in evaluating a large number of genotypes and measuring their tolerance to drought stress in the least time and efficiently. To avoid errors and obtain results as accurately as possible, it is recommended that one induvial score the traits as this method depends on the accuracy and skill of the individual who is recording the traits. Scoring both types of morphological traits (tolerance and recovery) are very important in understanding the different mechanism, as well as identifying the most promising genotypes that can tolerate and survive drought. It is also useful to study morphological traits in addition to the physiological traits because they provide us with different information that helps us to understand the changes that occur in the plants to know the different mechanisms that the plant uses to reduce the severity of drought stress in addition to enhancing the efficiency of selection. DTI was an effective tool and a very useful index for improving a group of traits and selecting the most drought-tolerant genotypes. The best ten drought-tolerant genotypes in this study are recommended to be evaluated in the field under drought conditions to test their yield attributes and then used for future breeding programs to produce wheat cultivars having more drought tolerance under Egyptian conditions. These genotypes have the highest accumulation of proline, glucose, total soluble sugars, and proteins concomitant with the lowest DTI. In addition, genetic analysis showed a high diversity among these genotypes in the number of tolerance genes present in each genotype. As these genotypes included eight genotypes from Egypt and two genotypes from other countries (Morocco and United States). Three candidate genotypes (MISR1, SAKHA93, and PI525434) for drought tolerance can be targeted in future breeding programs to increase diversity and genetic improvement of drought tolerance in wheat at early growth stages.

## Data Availability

The original contributions presented in the study are included in the article/Supplementary Material; further inquiries can be directed to the corresponding author.

## Abbreviations

WCC, Wheat core collection; TT, Tolerance traits; RT, Recovery traits; SL, Seedling length; LW, Leaf wilting; S_LW, Sum of leaf wilting; DTW, Days to wilting; DTR, Days to regrowth; RB, Regrowth biomass; DSR, Drought survival rate; TI, Tolerance index; RI, Recovery index; DTI, Drought tolerance index; P, Protein content; AM, Amino acids content; PRO, Proline content; G, Glucose content; F, Fructose content; TSC, Total soluble carbohydrates; DREB, Dehydration responsive element binding proteins; H2, Heritability; RDD, Reduction due to drought stress; IDD, Increases due to drought stress.
