# Supplementary material for: Genetic and morpho-physiological analyses of the tolerance and recovery mechanisms in seedling stage spring wheat under drought stress
Source: Front Genet. 2022 Oct 11;13:1010272. doi: 10.3389/fgene.2022.1010272 (PMC9593057; doi:10.3389/fgene.2022.1010272)
Supplement: Supplementary file 1 [file Presentation1.PPTX]

## Slide 1
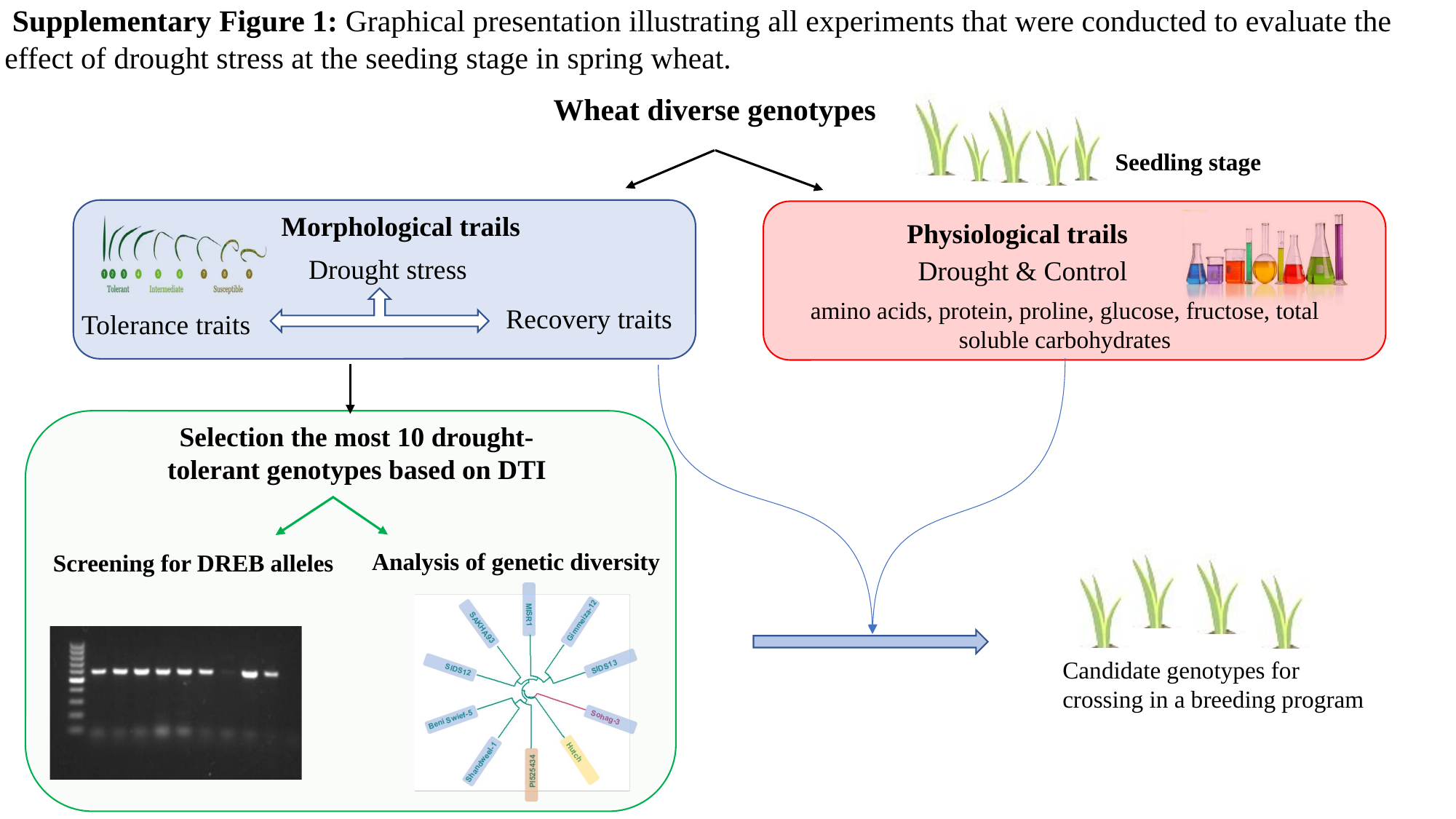

Supplementary Figure 1: Graphical presentation illustrating all experiments that were conducted to evaluate the effect of drought stress at the seeding stage in spring wheat.
Wheat diverse genotypes
Seedling stage
Morphological trails
Drought stress
Drought & Control
amino acids, protein, proline, glucose, fructose, total soluble carbohydrates
Recovery traits
Selection the most 10 drought- tolerant genotypes based on DTI
Candidate genotypes for crossing in a breeding program
Physiological trails
Tolerance traits
Analysis of genetic diversity
Screening for DREB alleles

## Slide 2
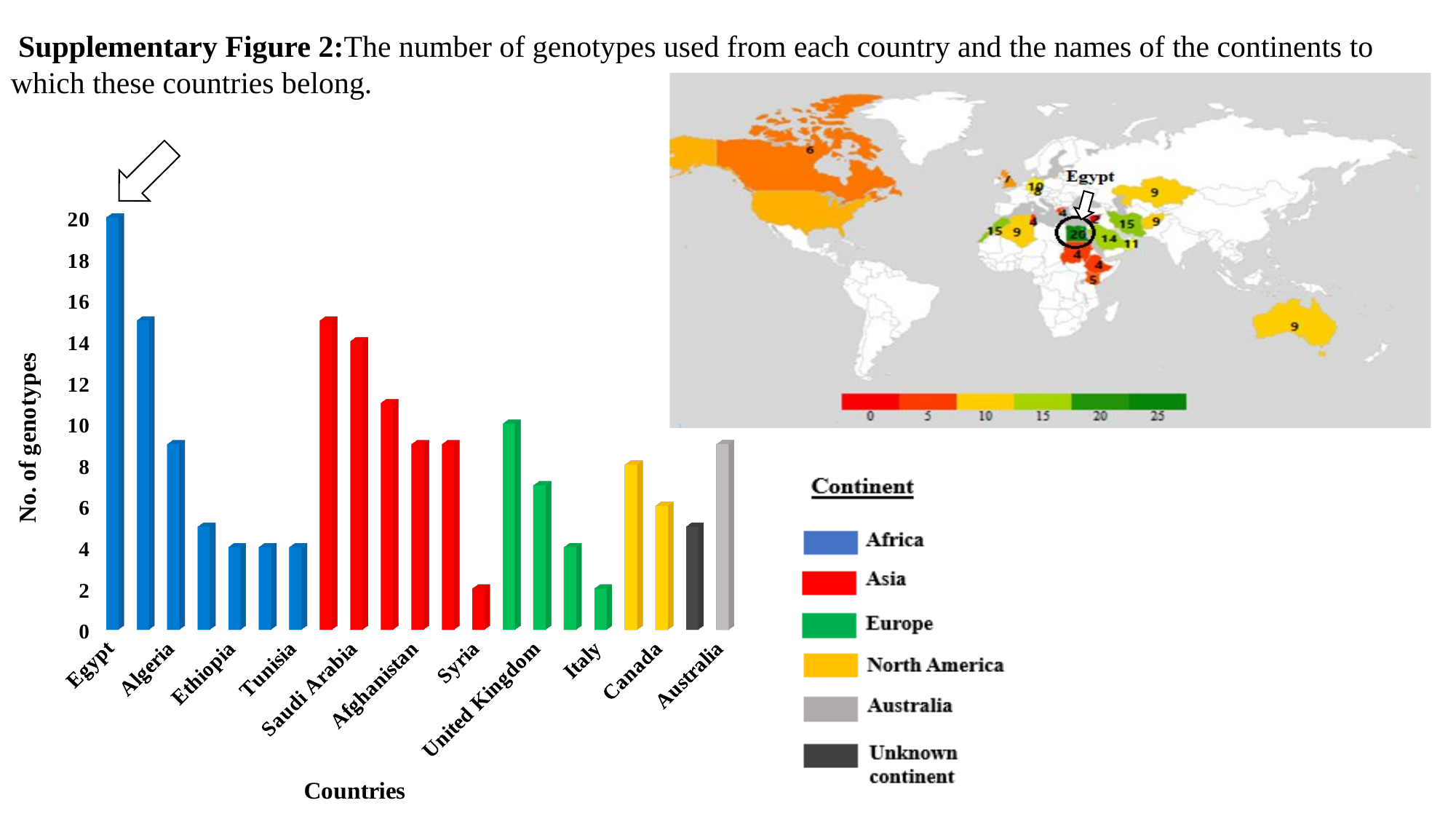

Supplementary Figure 2:The number of genotypes used from each country and the names of the continents to which these countries belong.
[unsupported chart]

## Slide 3
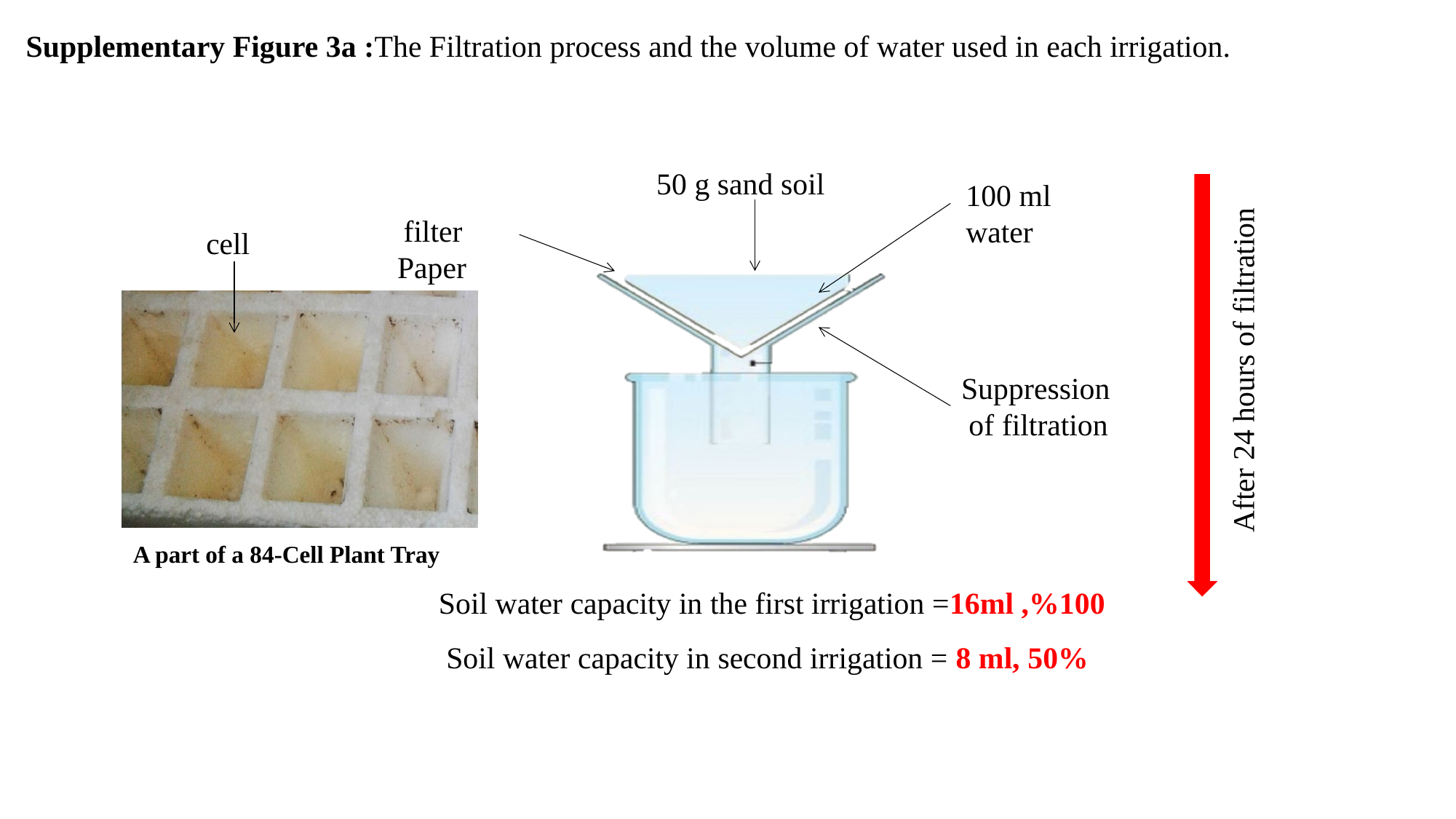

Supplementary Figure 3a :The Filtration process and the volume of water used in each irrigation.
50 g sand soil
100 ml water
 filter Paper
 cell
After 24 hours of filtration
Suppression of filtration
A part of a 84-Cell Plant Tray
Soil water capacity in the first irrigation =16ml ,%100
 Soil water capacity in second irrigation = 8 ml, 50%

## Slide 4
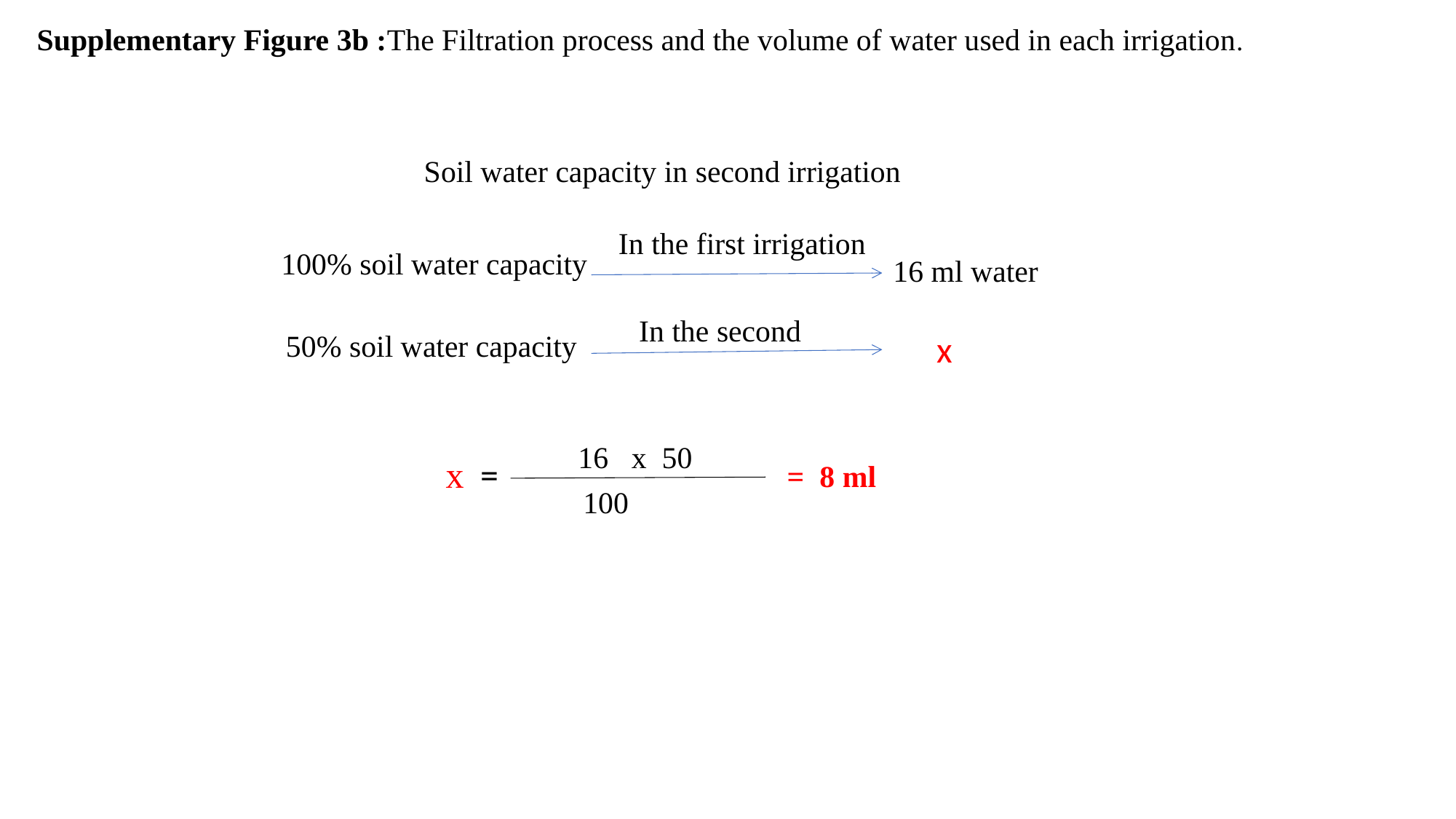

Supplementary Figure 3b :The Filtration process and the volume of water used in each irrigation.
Soil water capacity in second irrigation
In the first irrigation
100% soil water capacity
16 ml water
In the second
 x
50% soil water capacity
 16 x 50
x =
= 8 ml
 100

## Slide 5
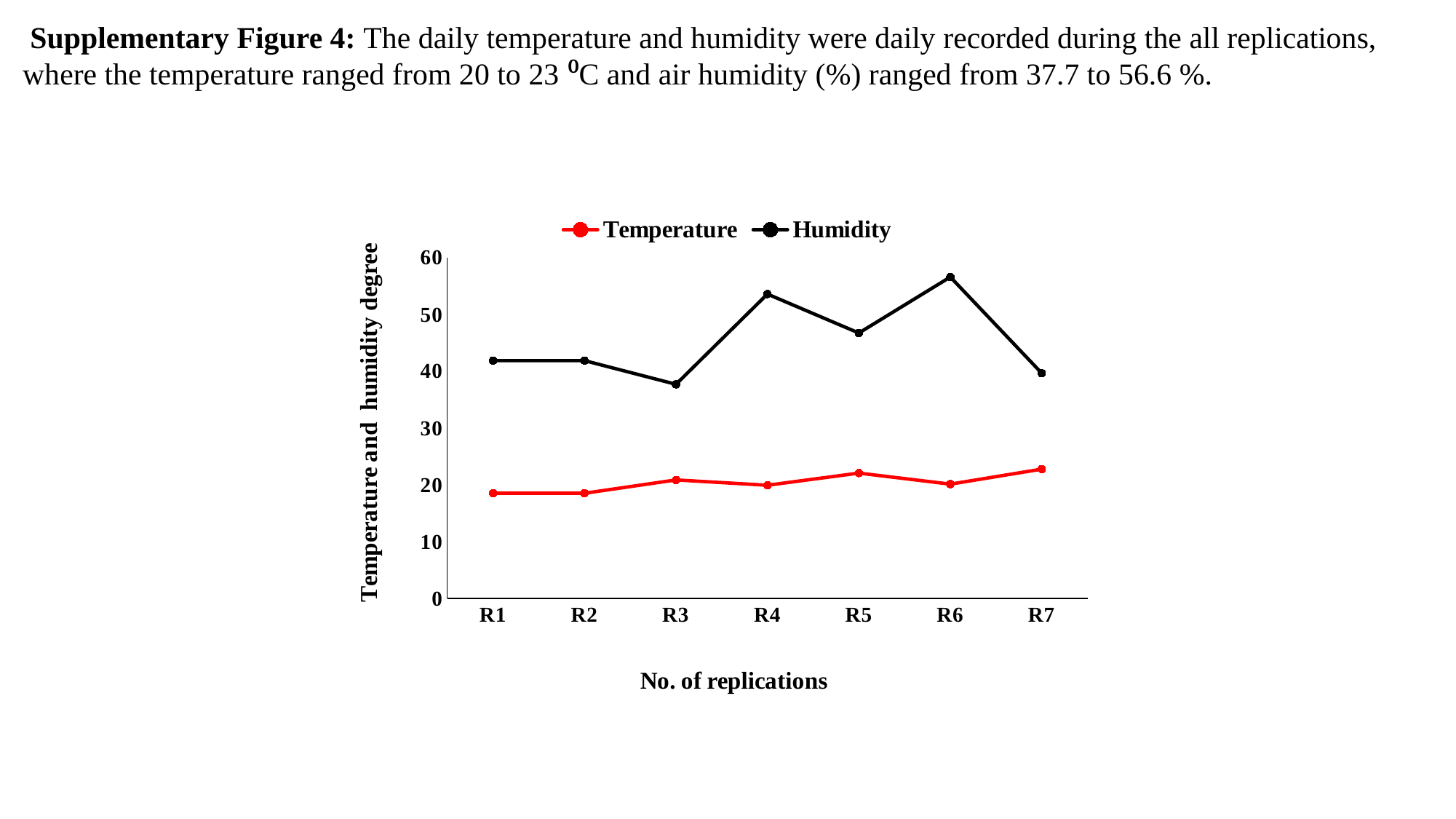

Supplementary Figure 4: The daily temperature and humidity were daily recorded during the all replications, where the temperature ranged from 20 to 23 ⁰C and air humidity (%) ranged from 37.7 to 56.6 %.
### Chart
| Category | Temperature | Humidity |
|---|---|---|
| R1 | 18.5 | 41.857142857142854 |
| R2 | 18.5 | 41.857142857142854 |
| R3 | 20.842105263157894 | 37.68421052631579 |
| R4 | 19.905263157894737 | 53.578947368421055 |
| R5 | 22.047368421052635 | 46.71578947368421 |
| R6 | 20.105263157894733 | 56.578947368421055 |
| R7 | 22.74736842105263 | 39.63157894736842 |

## Slide 6
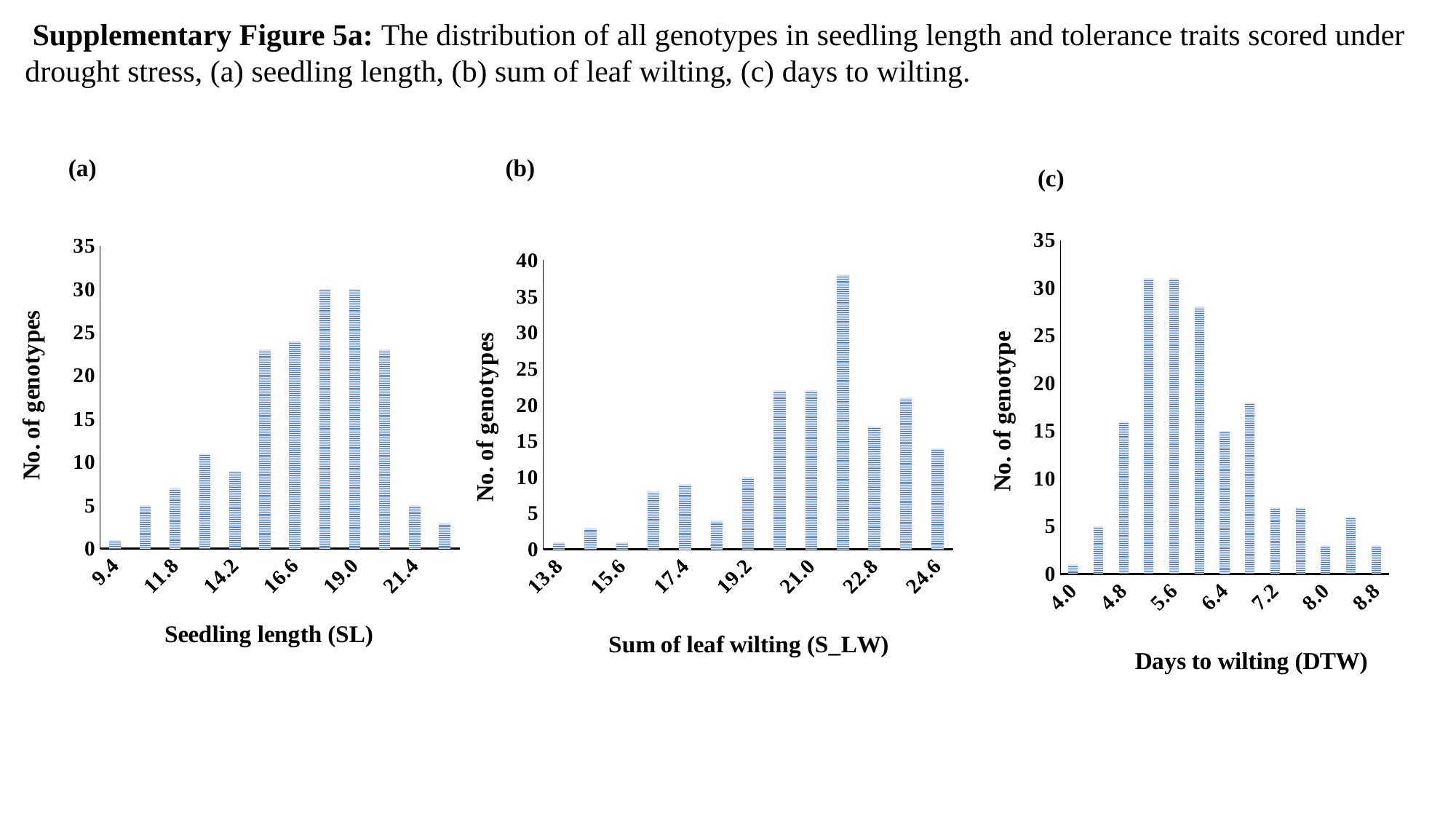

Supplementary Figure 5a: The distribution of all genotypes in seedling length and tolerance traits scored under drought stress, (a) seedling length, (b) sum of leaf wilting, (c) days to wilting.
(a)
(b)
(c)
### Chart
| Category | |
|---|---|
| 9.379999999999999 | 1.0 |
| 10.579999999999998 | 5.0 |
| 11.779999999999998 | 7.0 |
| 12.979999999999997 | 11.0 |
| 14.179999999999996 | 9.0 |
| 15.379999999999995 | 23.0 |
| 16.579999999999995 | 24.0 |
| 17.779999999999994 | 30.0 |
| 18.979999999999993 | 30.0 |
| 20.179999999999993 | 23.0 |
| 21.379999999999992 | 5.0 |
| 22.579999999999991 | 3.0 |
### Chart
| Category | |
|---|---|
| 4 | 1.0 |
| 4.4000000000000004 | 5.0 |
| 4.8000000000000007 | 16.0 |
| 5.2000000000000011 | 31.0 |
| 5.6000000000000014 | 31.0 |
| 6.0000000000000018 | 28.0 |
| 6.4000000000000021 | 15.0 |
| 6.8000000000000025 | 18.0 |
| 7.2000000000000028 | 7.0 |
| 7.6000000000000032 | 7.0 |
| 8.0000000000000036 | 3.0 |
| 8.4000000000000039 | 6.0 |
| 8.8000000000000043 | 3.0 |
### Chart
| Category | |
|---|---|
| 13.82 | 1.0 |
| 14.72 | 3.0 |
| 15.620000000000001 | 1.0 |
| 16.52 | 8.0 |
| 17.419999999999998 | 9.0 |
| 18.319999999999997 | 4.0 |
| 19.219999999999995 | 10.0 |
| 20.119999999999994 | 22.0 |
| 21.019999999999992 | 22.0 |
| 21.919999999999991 | 38.0 |
| 22.81999999999999 | 17.0 |
| 23.719999999999988 | 21.0 |
| 24.619999999999987 | 14.0 |

## Slide 7
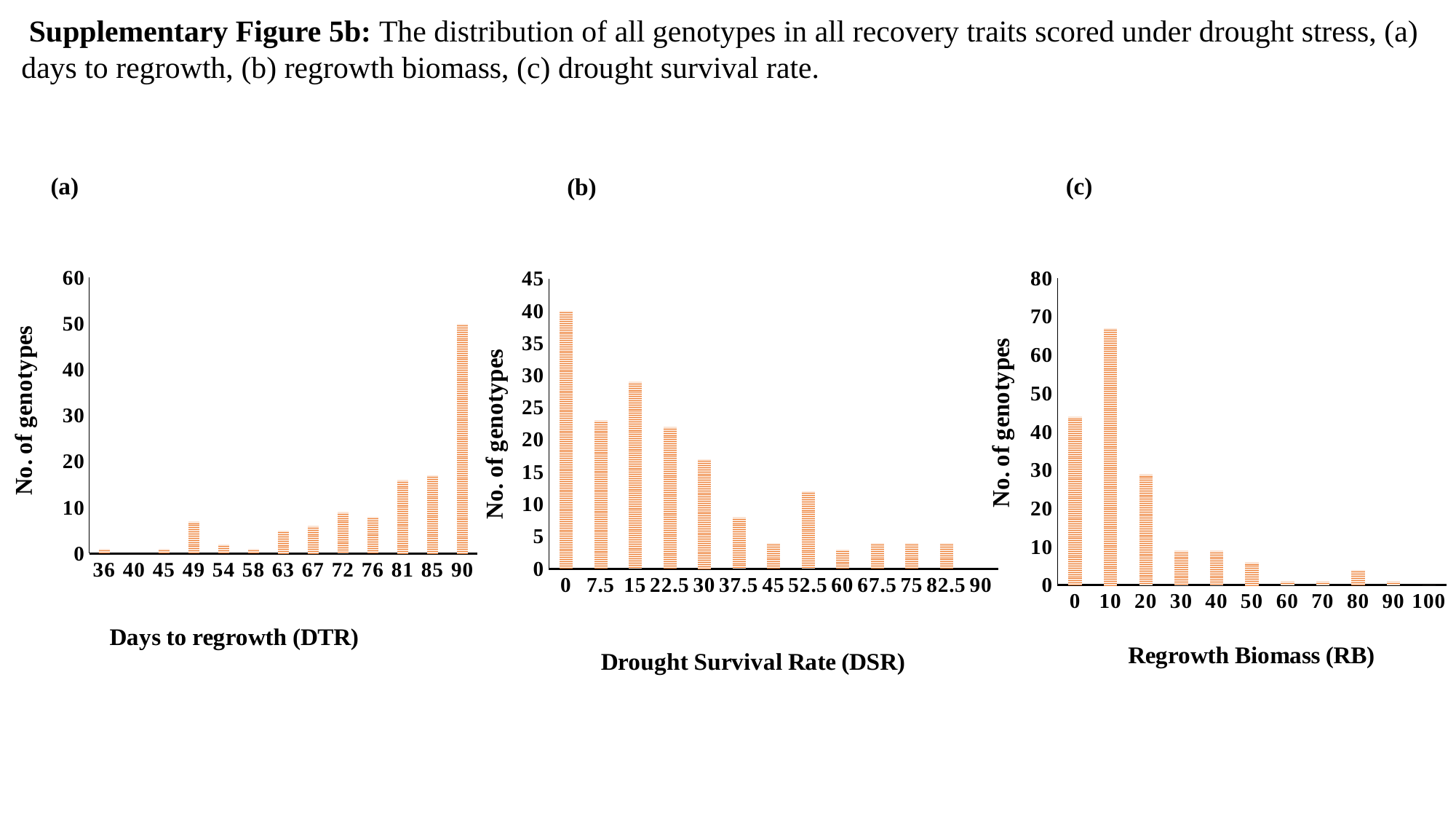

Supplementary Figure 5b: The distribution of all genotypes in all recovery traits scored under drought stress, (a) days to regrowth, (b) regrowth biomass, (c) drought survival rate.
(a)
(c)
(b)
### Chart
| Category | |
|---|---|
| 0 | 44.0 |
| 10 | 67.0 |
| 20 | 29.0 |
| 30 | 9.0 |
| 40 | 9.0 |
| 50 | 6.0 |
| 60 | 1.0 |
| 70 | 1.0 |
| 80 | 4.0 |
| 90 | 1.0 |
| 100 | 0.0 |
### Chart
| Category | |
|---|---|
| 35.67 | 1.0 |
| 40.17 | 0.0 |
| 44.67 | 1.0 |
| 49.17 | 7.0 |
| 53.67 | 2.0 |
| 58.17 | 1.0 |
| 62.67 | 5.0 |
| 67.17 | 6.0 |
| 71.67 | 9.0 |
| 76.17 | 8.0 |
| 80.67 | 16.0 |
| 85.17 | 17.0 |
| 89.67 | 50.0 |
[unsupported chart]

## Slide 8
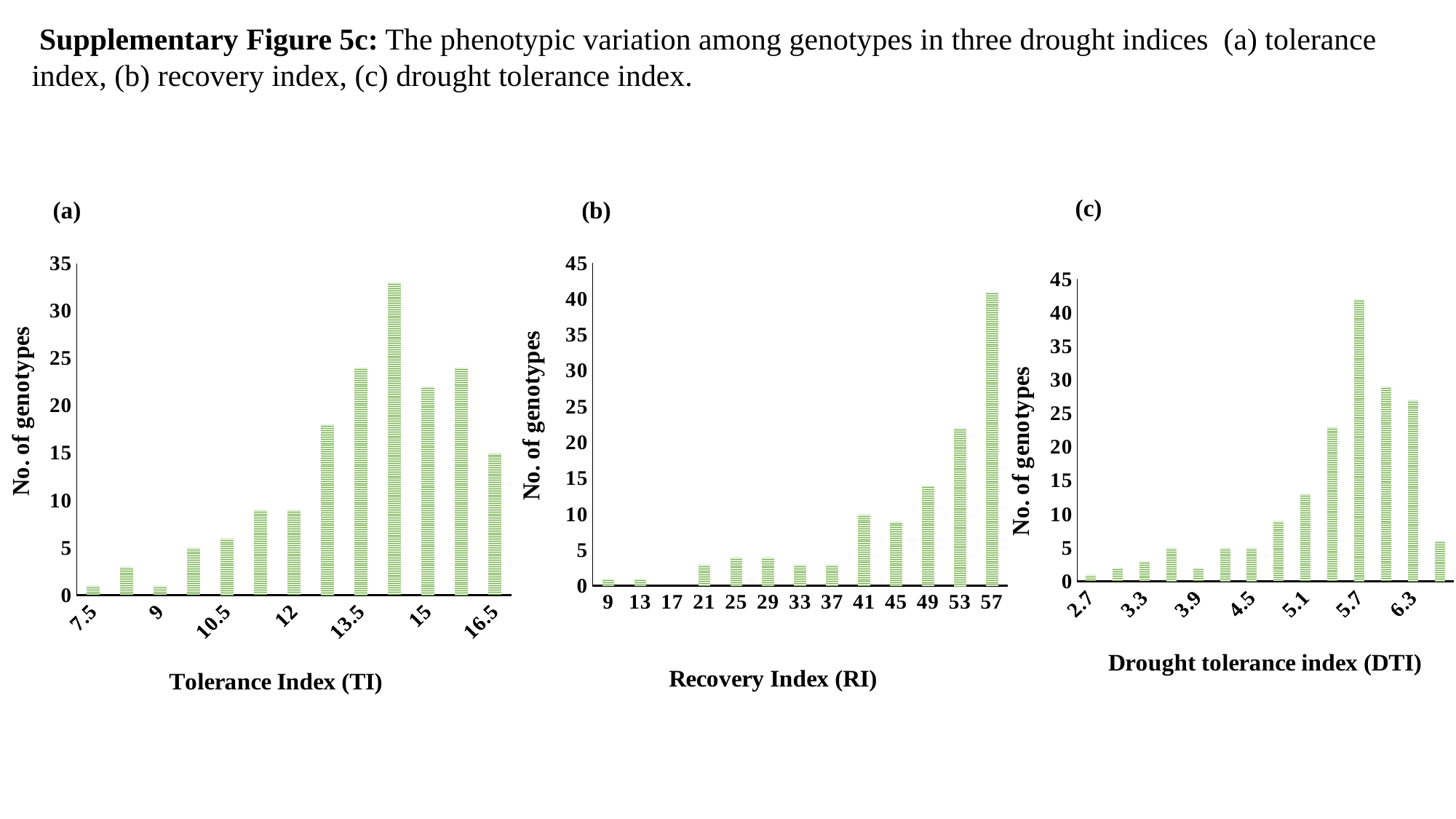

Supplementary Figure 5c: The phenotypic variation among genotypes in three drought indices (a) tolerance index, (b) recovery index, (c) drought tolerance index.
(c)
(a)
(b)
### Chart
| Category | |
|---|---|
| 9 | 1.0 |
| 13 | 1.0 |
| 17 | 0.0 |
| 21 | 3.0 |
| 25 | 4.0 |
| 29 | 4.0 |
| 33 | 3.0 |
| 37 | 3.0 |
| 41 | 10.0 |
| 45 | 9.0 |
| 49 | 14.0 |
| 53 | 22.0 |
| 57 | 41.0 |
### Chart
| Category | |
|---|---|
| 7.5 | 1.0 |
| 8.25 | 3.0 |
| 9 | 1.0 |
| 9.75 | 5.0 |
| 10.5 | 6.0 |
| 11.25 | 9.0 |
| 12 | 9.0 |
| 12.75 | 18.0 |
| 13.5 | 24.0 |
| 14.25 | 33.0 |
| 15 | 22.0 |
| 15.75 | 24.0 |
| 16.5 | 15.0 |
### Chart
| Category | |
|---|---|
| 2.7 | 1.0 |
| 3 | 2.0 |
| 3.3 | 3.0 |
| 3.5999999999999996 | 5.0 |
| 3.8999999999999995 | 2.0 |
| 4.1999999999999993 | 5.0 |
| 4.4999999999999991 | 5.0 |
| 4.7999999999999989 | 9.0 |
| 5.0999999999999988 | 13.0 |
| 5.3999999999999986 | 23.0 |
| 5.6999999999999984 | 42.0 |
| 5.9999999999999982 | 29.0 |
| 6.299999999999998 | 27.0 |
| 6.5999999999999979 | 6.0 |

## Slide 9
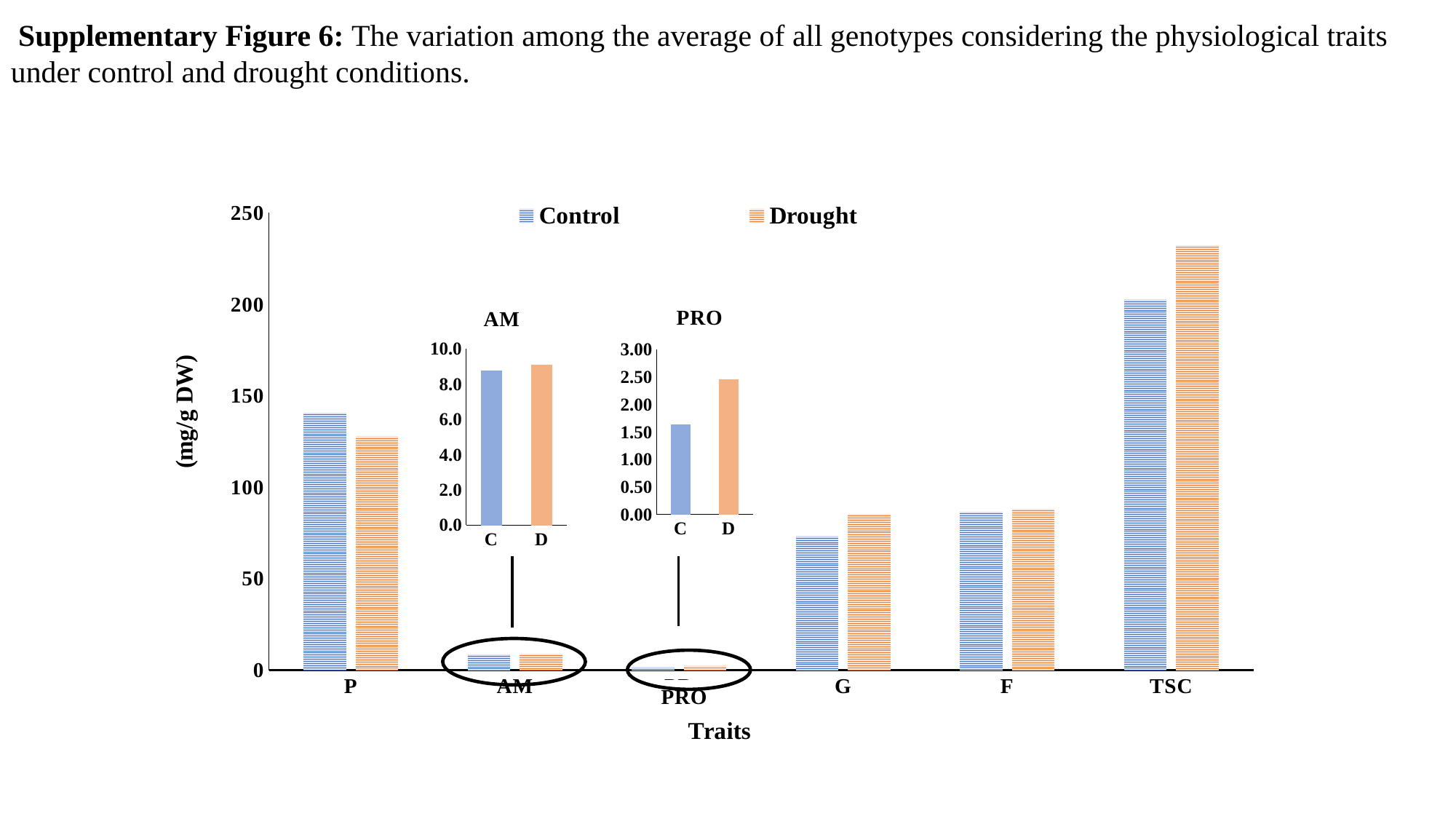

Supplementary Figure 6: The variation among the average of all genotypes considering the physiological traits under control and drought conditions.
### Chart
| Category | | |
|---|---|---|
| P | 140.68 | 127.74 |
| AM | 8.74 | 9.09 |
| PR | 1.63 | 2.45 |
| G | 73.33 | 85.47 |
| F | 86.69 | 88.17 |
| TSC | 202.55 | 232.31 |
### Chart: PRO
| Category | PR |
|---|---|
| C | 1.62630872483221 |
| D | 2.4495348837209288 |
### Chart:
| Category | AM |
|---|---|
| C | 8.74413173652694 |
| D | 9.090532544378696 |
